# Emergence of multidrug-resistant *Bacillus* spp. derived from animal feed, food and human diarrhea in South-Eastern Bangladesh

**DOI:** 10.1186/s12866-024-03199-3

**Published:** 2024-02-19

**Authors:** Md Atiqul Haque, Huilong Hu, Jiaqi Liu, Md Aminul Islam, Foysal Hossen, Md Arifur Rahman, Firoz Ahmed, Cheng He

**Affiliations:** 1 National Key Laboratory of Veterinary Public Health Security, College of Veterinary Medicine, China Agricultural University, Beijing, 100019 China; 2 Department of Microbiology, Faculty of Veterinary and Animal Science, Hajee Mohammad Danesh Science and Technology University, Dinajpur, 5200 Bangladesh; 3 Department of Microbiology, Faculty of Science, Noakhali Science and Technology University, Noakhali, 3814 Bangladesh

**Keywords:** Antimicrobial resistance, *Bacillus* spp., Food chain, Food security, Multidrug resistant, Resistant gene

## Abstract

**Background:**

Antimicrobial resistance poses a huge risk to human health worldwide, while Bangladesh is confronting the most severe challenge between the food supply and the huge consumption of antibiotics annually. More importantly, probiotics containing *Bacillus* spp. are claimed to be an alternative to antimicrobial stewardship programs. However, their antibiotic resistance remains elusive. Thus, we employed the antimicrobial susceptibility test and PCR to assess the prevalence of resistance, including multidrug resistance (MDR) and resito-genotyping of isolated *Bacillus* spp.

**Results:**

The phenotypic profile showed that *Bacillus* spp. were 100% sensitive to gentamicin (2 µg/mL), whereas lowered sensitivity to levofloxacin (67.8%, 0.5–1 µg/mL), ciprofloxacin (62.3%, 0.5–1 µg/mL), clindamycin (52.2%, 0.25–0.5 µg/mL), amoxicillin-clavulanic acid (37.6%, 0.06 µg/mL), azithromycin (33.4%, 1–2 µg/mL), tetracycline (25.6%, 2–4 µg/mL), nitrofurantoin (21.1%, 16–32 µg/mL), co-trimoxazole (19.2%, 2 µg/mL), and erythromycin (18.8%, 0.25–0.5 µg/mL). The strains were completely resistant to penicillin, amoxicillin-clavulanic acid, cefixime, ceftriaxone, vancomycin, and co-trimoxazole, and a species-specific trend was seen in both phenotypic and genotypic resistance patterns. Genotypic resistance indicated prevalence of the *bla1* (71.5%), *tetA* (33%), *erm1* (27%), *bla*_TEM_ (13.1%), *bla*_CTX-M-1_/*bla*_CTX-M-2_ /*sul1* (10.1%), *bla*_SHV_ (9.6%), and *qnrS* (4.1%) genes. The β-lactamase resistance gene *bla1* was found in all penicillin-resistant (MIC ≥ 32 µg/mL) *Bacillus* spp. One hundred ninety-one isolates (89.6%) were MDR, with 100% from diarrhea, 90.3% from food, and 88.7% from animal feed.

**Conclusion:**

Based on the MIC value and profile analysis of antibiotic resistance genes, this is the first study that *Bacillus* spp. antimicrobial susceptibilities have been identified in Bangladesh, and our study will shed light on the adverse effects of feed-borne *Bacillus* spp. emerging from animal feed to the food chain. A comprehensive investigation is urgently needed by policymakers on tolerance limits and harmful effects in the animal industry.

**Supplementary Information:**

The online version contains supplementary material available at 10.1186/s12866-024-03199-3.

## Background

Antimicrobial resistance (AMR) is a serious, multifaceted, and complicated healthcare concern worldwide that impacts people, animals, and the environment, resulting in harder-to-treat infections and even death. The “One Health” approach, which incorporates public health and veterinary regulators, the food and agriculture industry, financiers, environmentalists, and customers, is highlighted in the WHO-led Global Action Plan on Antimicrobial Resistance [1, 2]. AMR develops naturally over time, generally through genetic mutations that can transmit from one generation to another or between humans and animals via animal-sourced food. A variety of strategies, including target defence, target replacement, detoxification, and suppression of cellular antibiotic deposition, are used by bacteria to develop antimicrobial resistance (AMR) [2–4]. Although not all resistant bacteria produce diseases, they may initiate the manifestation of a disease or spread the gene encoding AMR to new bacterial pathogens in favorable environments [4]. Consequently, improper and abusive antibiotics might contribute to the development of different drug-resistant bacteria and can disperse antibiotic residues from various settings throughout the food supply chain, acting as a reservoir and propagation matrix for AMR with the potential for antibiotic-resistant gene (ARG) to cross the animal-to-human microbes due to bacterial contamination [5–7]. The transfer of ARGs is a common way that ABR spreads. Once resistant genes are transmitted by plasmids, transposons, or integrons, dispersion is quick, and horizontal gene transfer across bacteria is frequent. Hitherto, it was thought that this type of genetic exchange only occurred among the same bacterial species. Nevertheless, the transmission of ARGs among phylogenetically distinct bacterial clusters, particularly across gram-positive and gram-negative bacteria, has now been proven in natural habitats [8].

*Bacillus* spp. has long been used as probiotics in human, veterinary, aquaculture, plant, and environmental applications, either directly as microbial food or as food additives heavily contaminated in animal feed and food chains, making a major financial burden for livestock producers and a potential threat to public health [6, 9–11]. *B. cereus*-caused foodborne diseases are classified into diarrheal (toxico-infections) and emetic (intoxications) syndromes resulting from the formation of several toxins (enterotoxins such as *nhe*, *hbl*, *cytK*, *entFM*, *BceT*, *HlyII*; emetic toxins *ces*), which occur globally and are becoming a serious challenge [7, 12]. *B. cereus* exacerbates severe diarrhea and malnutrition in chickens and ducks by causing gizzard erosion and ulceration (GEU) and facilitating recurrent bacterial infections in the lungs by disrupting the gastrointestinal tract and following lung hemorrhagic lesions [13–16]. More interestingly, *B. cereus* was reported to induce non-gastrointestinal diseases, including bacteremia, septicemia, endophthalmitis, meningitis, endocarditis, urinary tract infections, and lung infections. Furthermore, *B. cereus* may lead to serious health effects, especially in newborn infants and immunosuppressed individuals [7, 12].

Nonetheless, some *B. cereus* and other bacteria possess ARG, which may spread among bacteria and ultimately impact humans through the food supply chain or the surroundings [5, 6]. Probiotic strains of *B. cereus*, *B. clausii*, *B. subtilis*, and *B. licheniformis* have shown resistance markers for β-lactams (*bla*_BCL-1_), chloramphenicol (*cat*_Bcl_), aminoglycosides (*aadD2*), macrolides (*erm34*), tetracycline (*tetM* and *tetK*), and erythromycin (*ermD* and *ermK*) [6]. The existence of mobile genetic components in *B. cereus* enables the uptake and transmission of drug-resistance genes from the environment [12].

Bangladesh, with a significant level of AMR and multidrug-resistant (MDR) bacteria against drugs indicated for use in both animals and people, confronts a local and worldwide hazard [3, 17–19]. However, *B. cereus* is resistant to numerous antibiotics, posing a global issue [20]. To inhibit the spread of AMR, it is essential to evaluate *Bacillus* spp. and their AMR profile. Some strains of *Bacillus* spp. are becoming increasingly resistant to antibiotics, allowing for the acquisition and emergence of new AMR strains. In our prior report, 39% of *Bacillus* spp. from animal feed and animal-based foods at a contamination level > 10^5^ CFU/g carried 80%, 71%, 55%, and 33% of the *entFM*, *cytK*, *nheABC,* and *hblACD* enterotoxin genes, respectively, and food-borne *Bacillus* spp. caused 4.5% of human diarrhea cases in south-eastern Bangladesh [15]. There is a lack of scientific data on the AMR in the livestock sector in Bangladesh. According to available report, resistant bacteria such as *E*. *coli*, *Salmonella* spp., *Klebsiella* spp., *Pseudomonas* spp., *Staphylococcus* spp., and *Vibrio* spp., were commonly detected in poultry, dairy cattle, raw milk, farm surroundings, and fish items [21, 22]. Nevertheless, there is a dearth of comprehensive data regarding the antibiotic resistance patterns of *Bacillus* spp. in the human, animal, and environmental sectors in Bangladesh. Thus, to fillin this knowledge gap, this work aimed to determine the prevalence of resistance, particularly MDR, and the correlated genetic factors in isolated *Bacillus* spp. In the present study, we focused on whether animal feed, food, and human stool could harbor MDR strains and disseminate them through the food supply chain.

## Results

### Bacterial isolates

The isolates tested in this investigation were chosen from our prior work [15] and identified to be *B. cereus*, *B. subtilis*, *B. amyloliquefaciens*, *B. licheniformis*, *B. thuringiensis*, *B. megaterium*, and *B. coagulans*, with 152, 56, and 10 strains from animal feed, food, and human diarrheal cases, respectively (Table S1, Figure S1-S4, Table S2-S3). In all analyzed samples, *B. cereus* was the predominant isolate (49.3–70%), followed by *B. subtilis* (14.2–30%), *B. amyloliquefaciens* (5.2–21.4%), *B. thuringiensis* (3.9–8.9%), *B. licheniformis* (8.5%), *B. megaterium* (5.2%), and *B. coagulans* (1.7–2.6%). Particularly, 7 *B*. *cereus* and 3 *B*. *subtilis* were isolated and identified from human stool with diarrhea cases.

### Phenotypic profile of antimicrobial resistance

MICs and MBCs of isolated bacteria were displayed in the Table 1. As for *B. cereus*, the MIC and MBC values were determined in our study: PG-NIT-CFM-CTR/VAN-TET/GEN/CMX-AZM-EM/CIP/LEV-CM-AMC and PG-NIT-CFM/CTR/VAN/CMX-TET/GEN-EM/CIP/LEV/CM-AZM-AMC respectively. While the MIC/MBC ratio for *B. subtilis* was as follows: NIT-CFM-VAN-CTR/TET/GEN/CMX-AZM-PG/CIP/LEV-EM/CM-AMC, *B. amyloliquefaciens* recorded a similar trend. In contrast, *B. licheniformis* and *B. thuringiensis* showed similar patterns: PG-NIT-CFM-VAN- CTR/TET/GEN/CMX-AZM/CIP-LEV/CM-EM-AMC. Interestingly, *B. megaterium* and *B. coagulans* followed the same pattern as NIT-TET/VAN-CTR/GEN/CMX-CFM/AZM- EM/CIP/LEV-PG/CM-AMC (Table 1).

**Table 1:** Antibiotic MICs and MBC of *Bacillus* species strains isolated from animal feed, food and diarrhea

Antibiotics	Bacterial strains →	*B. cereus*		*B. subtilis*		*B. amyloliquefaciens*		*B. licheniformis*		*B. thuringiensis*		*B. megaterium*		*B. coagulans*	
	MIC range tested (µg/mL)	MIC (µg/mL)	MBC (µg/mL)	MIC (µg/mL)	MBC (µg/mL)	MIC (µg/mL)	MBC (µg/mL)	MIC (µg/mL)	MBC (µg/mL)	MIC (µg/mL)	MBC (µg/mL)	MIC (µg/mL)	MBC (µg/mL)	MIC (µg/mL)	MBC (µg/mL)
PG	0.25–32	> 32	> 32	0.5	1	0.5	1	> 32	> 32	> 32	> 32	0.25	0.5	0.25	0.5
AMC	0.01–0.5	0.06	0.12	0.06	0.12	0.06	0.12	0.06	0.12	0.06	0.12	0.06	0.12	0.06	0.12
CFM	0.5–8	> 4	> 4	> 4	> 4	1	2	> 4	> 4	> 4	> 4	1	2	1	2
CTR	0.5–8	4	8	2	4	1	2	2	4	2	4	2	4	1	2
VAN	0.5–64	4	8	4	8	4	8	4	8	4	8	4	8	4	8
AZM	0.5–8	1	2	1	2	1	2	1	2	2	4	1	2	1	2
EM	0.25–32	0.5	1	0.25	0.5	0.25	0.5	0.25	1	0.5	1	0.5	1	0.5	1
TET	0.25–32	2	4	2	4	2	4	2	4	4	8	4	8	2	4
GEN	0.5–32	2	4	2	4	2	4	2	4	2	4	2	4	2	4
CM	0.25–8	0.25	1	0.25	1	0.25	0.5	0.5	1	0.5	1	0.25	0.5	0.5	1
NIT	1–128	16	32	16	32	16	32	16	32	32	64	16	32	16	32
CIP	0.12–16	0.5	1	0.5	1	0.5	1	1	2	1	2	0.5	1	0.5	1
LEV	0.12–16	0.5	1	0.5	1	0.5	1	0.5	1	1	2	0.5	1	0.5	1
CMX	1–128	2	8	2	4	2	4	2	4	2	4	2	4	2	4

*MIC* Minimum inhibitory concentration, *MBC* Minimum bactericidal concentration, *PG* Penicillin G, *AMC* Amoxicillin-Clavulanic acid, *CFM* Cefixime, *CTR* Ceftriaxone, *VAN* Vancomycin, *AZM* Azithromycin, *EM* Erythromycin, *TET* Tetracycline, *GEN* Gentamicin, *CM* Clindamycin, *NIT* Nitrofurantoin, *CIP* Ciprofloxacin, *LEV* Levofloxacin, *CMX* Co-Trimoxazole

**Fig. 1:**
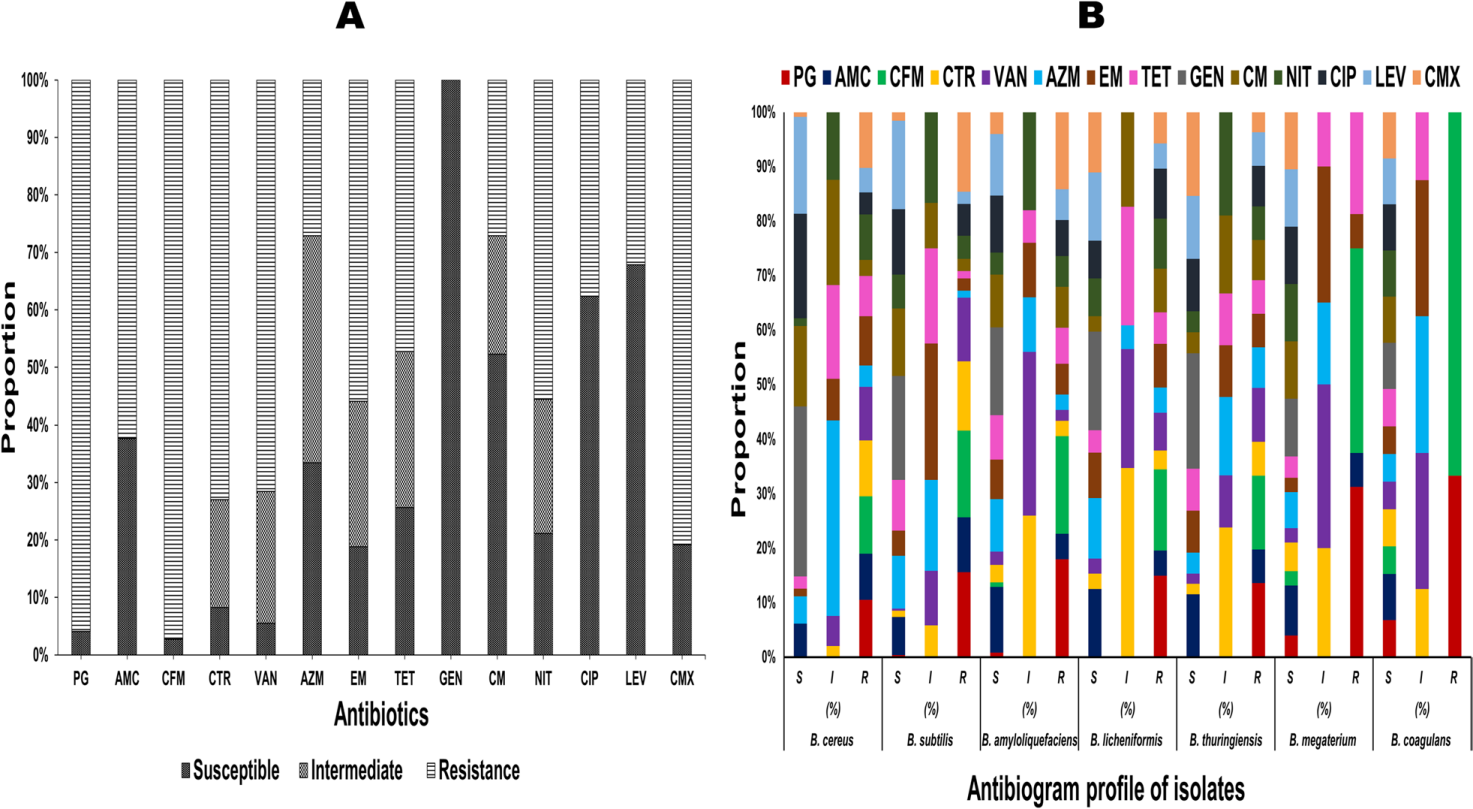
**A** Overall antibiogram profile of the isolated *Bacillus* spp. The bar diagram displayed the proportions of susceptible, intermediate and resistant strains among 218 isolated *Bacillus* strains to 14 antibiotics. PG: Penicillin G, AMC: Amoxicillin-Clavulanic acid, CFM: Cefixime, CTR: Ceftriaxone, VAN: Vancomycin, AZM: Azithromycin, EM: Erythromycin, TET: Tetracycline, GEN: Gentamicin, CM: Clindamycin, NIT: Nitrofurantoin, CIP: Ciprofloxacin, LEV: Levofloxacin, CMX: Co-Trimoxazole. **B** Overall antibiogram profile of 7 *Bacillus* strains

The antibiogram profiles of 14 antibiotics revealed that all isolated *Bacillus* spp. were generally sensitive to GEN (100%), LEV (67.8%), and CIP (62.3%), 52.2% to CM, 37.6% to AMC, and 33.4% to AZM (Table S4, Supplementary file 1). Further, the *Bacillus* spp. were generally resistant to β-lactam, glycopeptide, and sulfonamide antibiotics, including CFM (97.2%), PG (95.8%), CMX (81.1%), CTR (72.9%), VAN (71.5%), AMC (62.3%), while 55.9% were resistant to E, 55.5% to NIT, and 47.2% to TET (Fig. 1A, Table S4). The antibiotic-resistant pattern of AMC and AZM in diarrheal cases was significantly higher (*p* < 0.01) compared to animal feed and food samples. In contrast, the antibiotic-resistant pattern of CIP in animal feed was substantially greater (*p* < 0.05) than in food and diarrheal cases (Table 2).

**Table 2:** Antibiotic resistance pattern of *Bacillus* spp. in animal feed, food and diarrhea

Antibiotic	Antibiotic resistance pattern (%)			
	Animal feed	Food	Diarrhea	Level of significance
	% (n/N)	% (n/N)	% (n/N)	
PG	94.1 (143/152)	98.2 (55/56)	100 (10/10)	NS
AMC	55.2 (84/152)	87.5 (49/56)	100 (10/10)	**
CFM	95.3 (145/152)	100 (56/56)	100 (10/10)	NS
CTR	73.6 (112/152)	67.8 (38/56)	100 (10/10)	NS
VAN	69 (105/152)	73.2 (41/56)	100 (10/10)	NS
AZM	23 (35/152)	30.3 (17/56)	70 (7/10)	**
EM	53.9 (82/152)	60.7 (34/56)	70 (7/10)	NS
TET	48.6 (74/152)	39.2 (22/56)	70 (7/10)	NS
GEN	0	0	0	-
CM	23.6 (36/152)	37.5 (21/56)	20 (2/10)	NS
NIT	57.2 (87/152)	46.4 (26/56)	70 (7/10)	NS
CIP	44 (67/152)	33.9 (19/56)	0	*
LEV	33.5 (51/152)	23.2 (13/56)	50 (5/10)	NS
CMX	80.2 (122/152)	85.7 (48/56)	100 (10/10)	NS

*PG* Penicillin G, *AMC* Amoxicillin-Clavulanic acid, *CFM* Cefixime, *CTR* Ceftriaxone, *VAN* Vancomycin, *AZM* Azithromycin, *EM* Erythromycin, *TET* Tetracycline, *GEN* Gentamicin, *CM* Clindamycin, *NIT* Nitrofurantoin, *CIP* Ciprofloxacin, *LEV* Levofloxacin, *CMX* Co-Trimoxazole, *n* Number of resistant isolates, *N* Number of *Bacillus* isolates, * = significant (*p* < 0.05), ** = significant (*p* < 0.01), ns = non-significant

Regarding *B. cereus*, the isolates were generally sensitive to GEN (100%), CIP (61.6%), and LEV (57.1%) and generally resistant to PG/CFM (100%), CTR (97.4%), CMX (97.3%), VAN (98.2%), EM (85.7%), AMC (80.4%), NIT (79.4%), and TET (70.5%). In the instance of *B. subtilis*, the isolates were generally sensitive to GEN (100%), LEV (85.7%), CM (65.3%), CIP (63.3%), and AZM (51%). Furthermore, the *B. subtilis* isolates showed a somewhat similar resistant pattern to that of *B. cereus*. Interestingly, all *B. megaterium* and *B. coagulans* were 100% sensitive to GEN, CM, NIT, CIP, LEV, and CMX (Fig. 1B, Table S5). The ABR profiles of PG, AMC, CFM, CTR, VAN, AZM, EM, TET, CM, NIT, CIP, LEV, and CMX differed significantly (*p* < 0.01), and species-specific resistance was observed among the seven isolated *Bacillus* spp. (Table 3).

**Table 3:** Antibiotic resistant pattern of seven *Bacillus* species

Antibiotics	Fractions of resistant isolates							Level of significance
	*B. cereus*	*B. subtilis*	*B. amyloliquefaciens*	*B. licheniformis*	*B. thuringiensis*	*B. megaterium*	*B. coagulans*	
	% (n/N)	% (n/N)	% (n/N)	% (n/N)	% (n/N)	% (n/N)	% (n/N)	
PG	100 (112/112)	98 (48/49)	95 (19/20)	100 (13/13)	100 (11/11)	62.5 (5/8)	20 (1/5)	**
AMC	80.3 (90/112)	63.3 (31/49)	25 (5/20)	30.7 (4/13)	45.5 (5/11)	12.5 (1/8)	0	**
CFM	100 (112/112)	100 (49/49)	95 (19/20)	100 (13/13)	100 (11/11)	75.0 (6/8)	40 (2/5)	**
CTR	97.4 (109/112)	79.6 (39/49)	15 (3/20)	23.1 (3/13)	45.5 (5/11)	0	0	**
VAN	92.8 (104/112)	73.5 (36/49)	10 (2/20)	46.1 (6/13)	8/11 (72.7)	0	0	**
AZM	37.5 (42/112)	8.2 (4/49)	15 (3/20)	30.8 (4/13)	54.5 (6/11)	0	0	**
EM	85.7 (96/112)	14.3 (7/49)	30 (6/20)	53.8 (7/13)	45.5 (5/11)	12.5 (1/8)	0	**
TET	70.5 (79/112)	8.2 (4/49)	35 (7/20)	38.5 (5/13)	45.5 (5/11)	37.5 (3/8)	0	**
GEN	0	0	0	0	0	0	0	**-**
CM	27.7 (31/112)	14.3 (7/49)	40 (8/20)	53.8 (7/13)	54.5 (6/11)	0	0	**
NIT	79.4 (89/112)	26.5 (13/49)	30 (6/20)	61.5 (8/13)	45.5 (5/11)	0	0	**
CIP	38.4 (43/112)	36.7 (18/49)	35 (7/20)	61.5 (8/13)	54.5 (6/11)	0	0	*
LEV	42.8 (48/112)	14.3 (7/49)	35 (7/20)	30.8 (4/13)	54.5 (6/11)	0	0	**
CMX	97.3 (109/112)	91.8 (45/49)	75 (15/20)	61.5 (8/13)	27.3 (3/11)	0	0	**

*PG* Penicillin G, *AMC* Amoxicillin-Clavulanic acid, *CFM* Cefixime, *CTR* Ceftriaxone, *VAN* Vancomycin, *AZM* Azithromycin, *EM* Erythromycin, *TET* Tetracycline, *GEN* Gentamicin, *CM* Clindamycin, *NIT* Nitrofurantoin, *CIP* Ciprofloxacin, *LEV* Levofloxacin, *CMX* Co-Trimoxazole. *n* Number of resistant isolates, *N* Number of *Bacillus* isolates, *significant (*p* < 0.05), **significant (*p* < 0.01)

### Pearson correlation coefficients (*ρ*) for pairs of antibiotics to assess ABR *Bacillus* isolates

Bivariate analysis showed a highly significant association (*p* =  < 0.001–0.000) between the resistance patterns of TET and PG, EM/NIT and CFM, AZM/CM and AMC, NIT/CFM/CTR/VAN/EM/CMX and PG, CTR/VAN/EM/TET/NIT/CIP/LEV/CMX and AMC, CTR/VAN/CMX and CFM, VAN/AZM/EM/TET/CM/NIT/CIP/LEV/CMX and CTR, AZM/EM/TET/CM/NIT/CIP/LEV/CMX and VAN, EM/TET/NIT/CIP/CMX and AZM, TET/CM/CIP/LEV/CMX and EM, CM/NIT/CIP/LEV/CMX and TET, NIT/CIP/LEV/CMX/ and CM, CIP/LEV/CMX and NIT, LEV/CMX and CIP, CMX and LEV, and NIT and EM, CM and AZM. A moderate association (*p* = 0.035–0.017) was found between AMC/CIP/LEV and PG, and TET and CFM. There was a weaker correlation (*p* > 0.05) between AZM/CM/AMC/CIP/LEV and CFM, and AMC/AZM and PG (Table 4).

**Table 4:** Pearson correlation coefficients for pairs of antibiotics to assess antibiotic-resistant *Bacillus* isolates from animal feed, food and diarrhea

Statistical analysis		PG	AMC	CFM	CTR	VAN	AZM	EM	TET	GEN	CM	NIT	CIP	LEV	CMX
PG	Pearson Correlation Coefficient	1													
	*p*-value (two tailed)	-													
AMC	Pearson Correlation Coefficient	0.148*	1												
	*p*-value (two tailed)	0.029													
CFM	Pearson Correlation Coefficient	0.811**	0.120	1											
	*p*-value (two tailed)	< 0.001	0.077												
CTR	Pearson Correlation Coefficient	0.341**	0.434**	0.276**	1										
	*p*-value (two tailed)	< 0.001	< 0.001	< 0.001											
VAN	Pearson Correlation Coefficient	0.329**	0.450**	0.267**	0.966**	1									
	*p*-value (two tailed)	< 0.001	< 0.001	< 0.001	< 0.001										
AZM	Pearson Correlation Coefficient	0.126	0.190**	0.102	0.371**	0.384**	1								
	*p*-value (two tailed	0.062	0.005	0.131	< 0.001	< 0.001									
EM	Pearson Correlation Coefficient	0.234**	0.473**	0.190**	0.687**	0.711**	0.540**	1							
	*p*-value (two tailed	< 0.001	< 0.001	0.005	< 0.001	< 0.001	< 0.001								
TET	Pearson Correlation Coefficient	0.196**	0.379**	0.159*	0.576**	0.597**	0.644**	0.840**	1						
	*p*-value (two tailed	0.004	< 0.001	0.019	< 0.001	< 0.001	< 0.001	< 0.001							
GEN	Pearson Correlation Coefficient	-^c^	-^c^	-^c^	-^c^	-^c^	-^c^	-^c^	-^c^	-^c^					
	*p*-value (two tailed	-	-	-	-	-	-	-	-	-					
CM	Pearson Correlation Coefficient	0.126	0.190**	0.102	0.371**	0.384**	1.000**	0.540**	0.644**	-^c^	1				
	*p*-value (two tailed	0.062	0.005	0.131	< .001	< .001	0.000	< .001	< .001	-^c^					
NIT	Pearson Correlation Coefficient	0.232**	0.468**	0.188**	0.680**	0.704**	0.545**	0.991**	0.847**	-^c^	0.545**	1			
	*p*-value (two tailed	< .001	< .001	0.005	< .001	< .001	< .001	< .001	< .001	-^c^	< .001				
CIP	Pearson Correlation Coefficient	0.161*	0.288**	0.131	0.473**	0.490**	0.784**	0.689**	0.820**	-^c^	0.784**	0.695**	1		
	*p*-value (two tailed	0.017	< .001	0.054	< .001	< .001	< .001	< .001	< .001	-^c^	< .001	< .001			
LEV	Pearson Correlation Coefficient	0.143*	0.237**	0.166	0.419**	0.434**	0.886**	0.610**	0.727**	-^c^	0.886**	0.616**	0.886**	1	
	*p*-value (two tailed	0.035	< .001	0.088	< .001	< .001	< .001	< .001	< .001	-^c^	< .001	< .001	< .001		
CMX	Pearson Correlation Coefficient	0.431**	0.343**	0.350**	0.790**	0.763**	0.293**	0.543**	0.455**	-^c^	0.293**	0.538**	0.374**	0.331**	1
	*p*-value (two tailed	< .001	< .001	< .001	< .001	< .001	< .001	< .001	< .001	-^c^	< .001	< .001	< .001	< .001	-

^*^Correlation is significant at the 0.05 level (2-tailed), ** Correlation is significant at the 0.01 level (2 tailed), ^C^Cannot be computed because at least one of the variables is constant, *PG* Penicillin G, *AMC* Amoxicillin-Clavulanic acid, *CFM* Cefixime, *CTR* Ceftriaxone, *VAN* Vancomycin, *AZM* Azithromycin, *EM* Erythromycin, *TET* Tetracycline, *GEN* Gentamicin, *CM* Clindamycin, *NIT* Nitrofurantoin, *CIP* Ciprofloxacin, *LEV* Levofloxacin, *CMX* Co-Trimoxazole

### Genotyping profile of antibiotic resistance

**Fig. 2:**
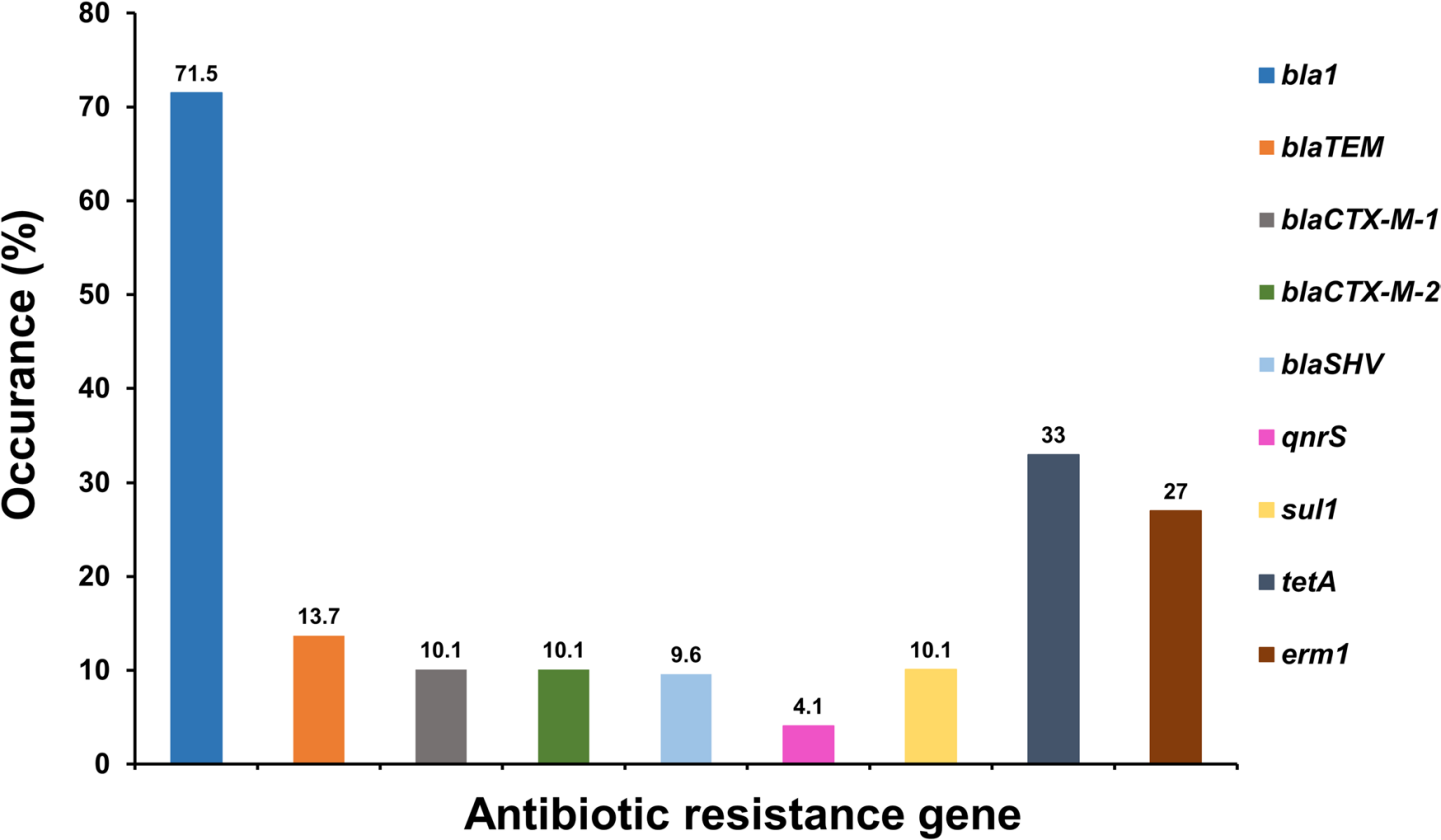
Distribution of antibiotic resistance genes of *Bacillus* spp. isolated from animal feed, food and diarrheal cases in Bangladesh. The numerical value displayed above each bar shows the positive rate associated with the respective antibiotic resistance genes

**Fig. 3:**
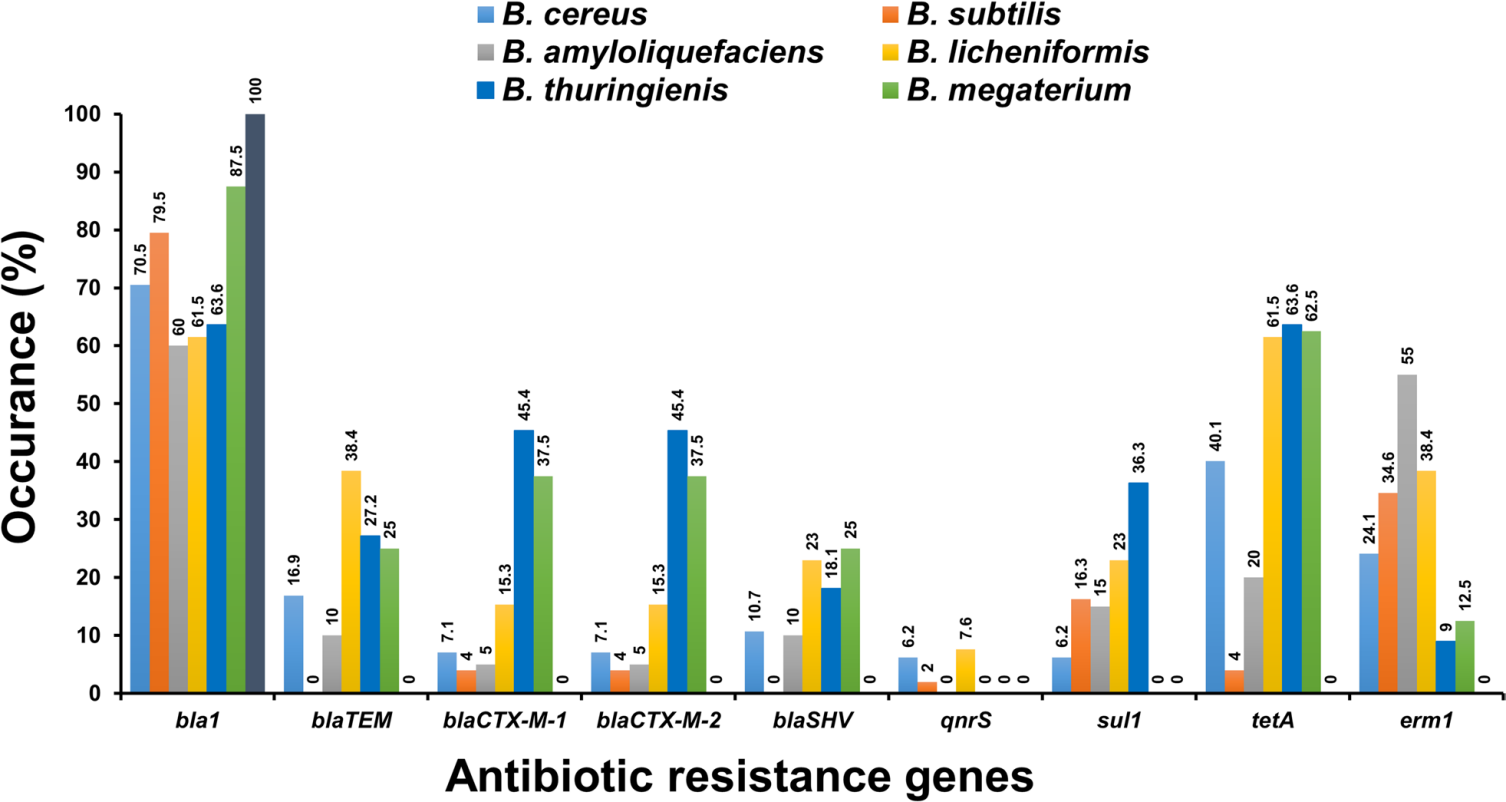
Species wise distribution of antibiotic resistance gene of *Bacillus* spp. isolated from animal feed, food and diarrheal cases in Bangladesh. The numerical value displayed above each specific color bar shows the positive rate associated with antibiotic resistance genes of the respective *Bacillus* strains

**Fig. 4:**
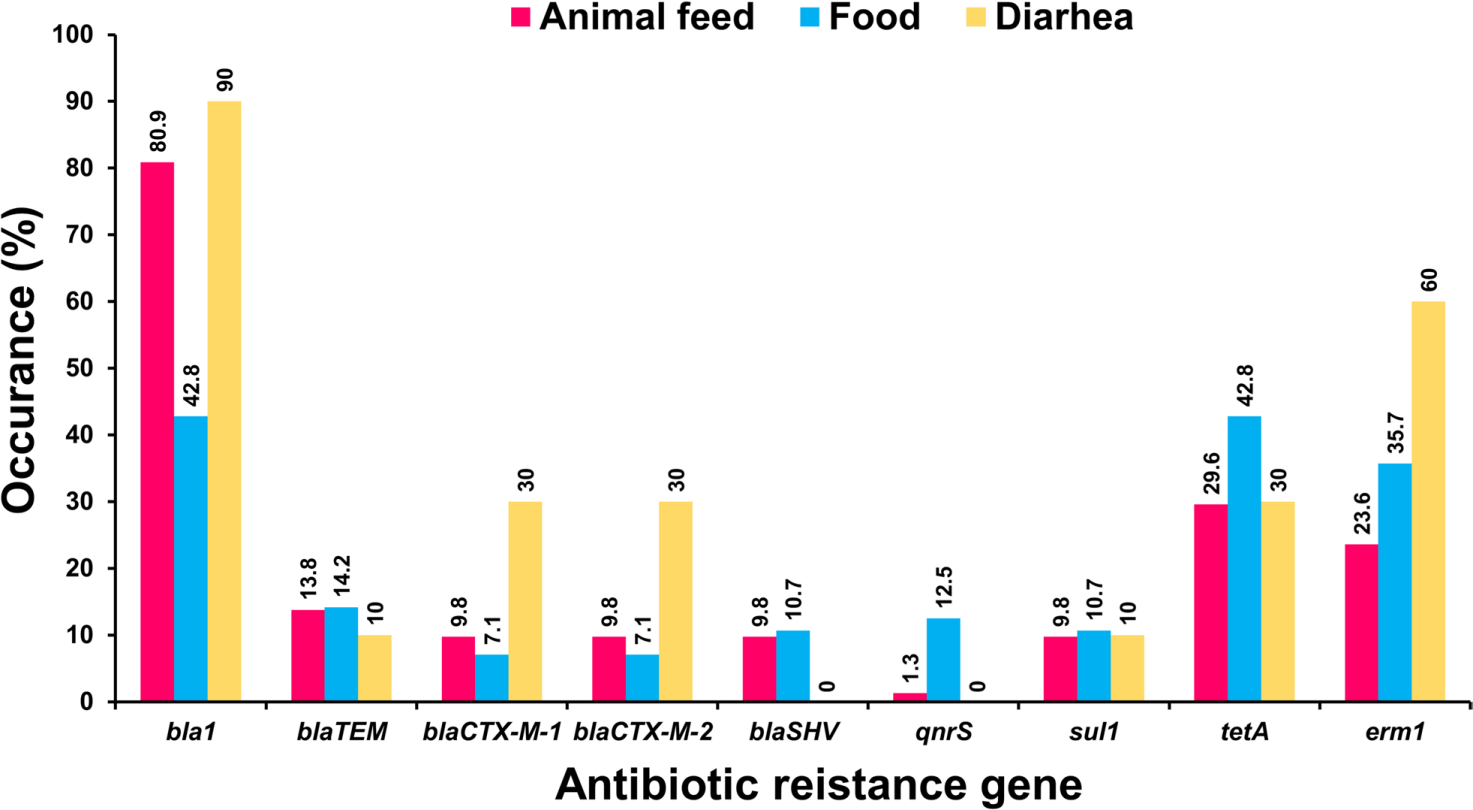
Sample wise distribution of antibiotic resistance gene of *Bacillus* spp. isolated from animal feed, food and diarrheal cases in Bangladesh. The numerical value displayed above each specific color bar shows the positive rate associated with respective antibiotic resistance genes in animal feed, food and diarrhea

Antibiotic resistance genes (ARGs) of isolated *Bacillus* spp. showed unique amplified target genes (Fig. S5A-S5I). The ARG profiles exhibited by *Bacillus* spp. were categorized into ten discrete types of isolates, indicating a considerable degree of genetic heterogeneity (Table 5). Of the 9 ARGs, the *bla1* gene was the most frequent (71.5%), followed by *tetA* (33%), *erm1* (27%), *bla*_TEM_ (13.7%), *bla*_SHV_ (9.6%), and *qnrS* (4.1%). The *bla*_CTX-M-1_, *bla*_CTX-M-2_, and *sul1* genes were observed with an equivalent frequency of 10.1% (Fig. 2, Table S6). According to sample-wise distribution, the prevalence of *bla1* was highest (80.9%) and *qnrS* was lowest (1.3%) in animal feed, whereas *bla1* and *tetA* were highest (42.8%) and *bla*_CTX-M-1_ and *bla*_CTX-M-2_ were lowest (7.1%) in food, and *bla1* was highest (90.0%) and *bla*_TEM_ and *sul1* were lowest (10.0%) in diarrheal cases. The *bla1* and *erm1* genes were significantly higher in the diarrheal case (*p* < 0.01 and *p* < 0.05, respectively) compared to the animal feed and food samples. In contrast, the *qnrS* gene was significantly greater (*p* < 0.01) in animal feed compared to both food and diarrheal cases (Table S7). The distribution of ARGs among 7 *Bacillus* strains is shown in Fig. 3 and Table S8. Obviously, *bla1* and *tetA* were predominantly distributed among *Bacillus* isolates, accounting for 40.1–100%. Furthermore, *B. cereus* and *B. licheniformis* harbored the remaining ARGs (*bla*_TEM_, *bla*_CTX-M-1_, *bla*_CTX-M-2_, *bla*_SHV_, *qnrS*, *sul1,* and *erm1*) at a rate of 6.2–38.4%. Interestingly, *B. coagulans* lacked almost all of the ARGs except for the *bla1* gene. The distribution of ARGs among animal feed, food and diarrheal cases is depicted in Fig. 4 and Table S9. Importantly, there were substantial variations in the prevalence of 6 ARGs among 7 isolated *Bacillus* spp., including *bla*_TEM_/*bla*_CTX-M-1_/*bla*_CTX-M-2_/*tetA* (*p* < 0.01), *sul1/erm1* (*p* < 0.05), and species-specific occurrence was observed (Table S8).

**Table 5:** Antibiotic resistance gene profiling by PCR

Profile	Antibiotic resistance gene									Origin								Total (%) (*n* = 218)
	*bla1*	*bla*_TEM_	*bla*_CTX-M-1_	*bla*_CTX-M-2_	*bla*_*S*HV_	*qnr*S	*sul1*	*tetA*	*erm1*	Animal feed					Food		Diarrhea	
										LF (*n* = 42)	BF (*n* = 37)	DF (*n* = 26)	CF (*n* = 28)	FF (*n* = 19)	E (*n* = 25)	M (*n* = 31)	HS (*n* = 10)	
I	+	+	+	+	-	-	+	+	+	1	1	2	2	1	1	1	1	10 (4.6)
II	+	+	-	-	+	-	-	+	-	6	2	4	1	2	3	3		21 (9.6)
III	+	-	+	+	-	-	-	+	-	3	3	1	1	1	1	1	2	13 (5.9)
IV	+	-	-	-	-	-	-	-	+	10	4	9	3	5		3	4	38 (17.4)
V	+	-	-	-	-	-	-	-	-	21	19	10	8	5	2	9	2	76 (34.8)
VI	-	-	-	-	-	-	-	+	+						5	5		10 (4.5)
VII	-	-	-	-	-	-	-	+	-	1	1		10	3		4		19 (8.7)
VIII	-	-	-	-	-	+	-	-	-		1			1	5	2		9 (4.1)
IX	-	-	-	-	-	-	-	-	+		1				3	1	1	6 (2.7)
X	-	-	-	-	-	-	+	-	-		5		3	1	5	2		16 (7.3)

*LF* Layer feed, *BF* Broiler feed, *DF* Duck feed, *CF* Cattle feed, *FF* Fish feed, *E* Egg, *M* Milk, *HS* Human stool, + positive, -negative

### Pearson correlation coefficients (*ρ*) for pairs of ARGs of *Bacillus* isolates

A bivariate analysis conducted on ARGs of *Bacillus* isolates showed a highly significant correlation (*p* =  < 0.001–0.000) between *bla1* and *bla*_SHV_/*bla*_CTX-M-1_/*bla*_CTX-M-2_, *sul1* and *bla*_TEM_, *sul1* and *bla*_SHV_, *bla1* and *bla*_TEM_/*tetA*/*erm1*, *bla*_TEM_ and *bla*_CTX-M-1_/*bla*_CTX-M-2_/*bla*_SHV_/*tetA/erm1*, *bla*_CTX-M-1_ and *sul1/tetA/erm1*, *bla*_CTX-M-2_ and *bla*_SHV_/*sul1/tetA/erm1*, *bla*_SHV_ and *tetA/erm1*, and *erm1* and *tetA*, *bla*_CTX-M-1_ and *bla*_CTX-M-2_. A moderate association (*p* = 0.027–0.015) was observed between *qnrS/sul1* and *bla1*, *tetA/erm1,* and *sul1*. There were weaker correlations (*p* > 0.05) between *qnrS* and *sul1/bla*_TEM_/*erm1/bla*_CTX-M-_1/*bla*_CTX-M-2_/*bla*_SHV_/ *tetA* (Table 6).

**Table 6:** Pearson correlation coefficients for pairs of ARGs of *Bacillus* isolates from animal feed, food and diarrhea

Statistical analysis		*bla1*	*bla*_*TEM*_	*bla*_*CTX-M-1*_	*bla*_*CTX-M-2*_	*bla*_*SHV*_	*qnrS*	*sul1*	*tetA*	*erm1*
*bla1*	Pearson Correlation Coefficient	1								
	*p*-value (two tailed)	-								
*bla*_*TEM*_	Pearson Correlation Coefficient	0.252**	1							
	*p-*value (two tailed)	< 0.001	-							
*bla*_*CTX-M-1*_	Pearson Correlation Coefficient	0.211**	0.839**	1						
	*p*-value (two tailed)	0.002	< 0.001	-						
*bla*_*CTX-M-2*_	Pearson Correlation Coefficient	0.211**	0.839**	1.000**	1					
	*p*-value (two tailed)	0.002	< 0.001	0.000	-					
*bla*_*SHV*_	Pearson Correlation Coefficient	0.206**	0.817**	0.975**	0.975**	1				
	*p*-value (two tailed)	0.002	< 0.001	< 0.001	< 0.001	-				
*qnrS*	Pearson Correlation Coefficient	0.165*	0.092	0.096	0.096	0.097	1			
	*p*-value (two tailed	0.015	0.176	0.157	0.157	0.153	-			
*sul1*	Pearson Correlation Coefficient	0.150*	0.208**	0.239**	0.239**	0.233**	0.016	1		
	*p-*value (two tailed	0.026	0.002	< 0.001	< 0.001	< 0.001	0.815	-		
*tetA*	Pearson Correlation Coefficient	0.443**	0.569**	0.477**	0.477**	0.465**	0.098	0.150*	1	
	*p*-value (two tailed	< 0.001	< 0.001	< 0.001	< 0.001	< 0.001	0.151	0.027	-	
*erm1*	Pearson Correlation Coefficient	0.384**	0.656**	0.550**	0.550**	0.536**	0.093	0.159*	0.867**	1
	*p*-value (two tailed	< 0.001	< 0.001	< 0.001	< 0.001	< 0.001	0.169	0.019	< 0.001	-

*ARGs* Antibiotic resistance genes, *Correlation is significant at the 0.05 level (2-tailed), **Correlation is significant at the 0.01 level (2 tailed)

### MDR and MAR resistance profiles of *Bacillus* spp.

**Fig. 5:**
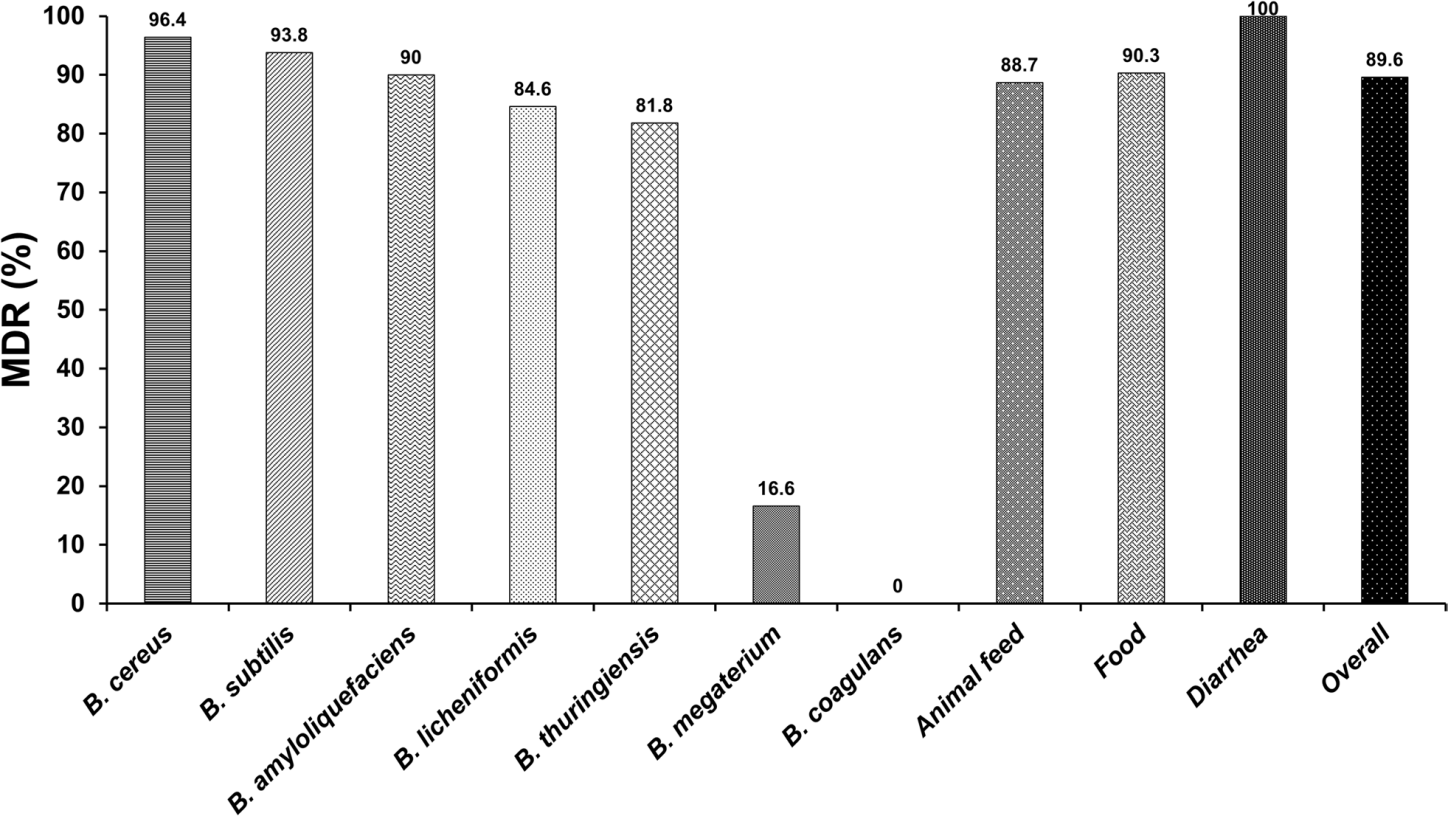
Distributions of multidrug resistant pattern in isolated *Bacillus* spp., animal feed, food, diarrhea and overall. The numerical value displayed above each specific bar shows the positive rate of the respective multidrug resistance pattern

Antibiogram typing revealed that 89.6% of isolated *Bacillus* strains were MDR (Fig. 5). *B. cereus* exhibited a higher MDR (96.4%), followed by *B. subtilis* (93.8%), *B. amyloliquefaciens* (90%), *B. licheniformis* (84.6%), *B. thuringiensis* (81.8%), and *B. megaterium* (16.6%), while *B. coagulans* had no MDR. Moreover, MDR was found in 100% of diarrheal isolates, followed by food (90.3%) and animal feed (88.7%) isolates. Interestingly, the species-wise MDR patterns differed significantly (*p* < 0.01); in contrast, the sample-wise distribution was not statistically different (*p* > 0.05) (Table 7, Table S10). Among the antibiogram types, pattern PG-AMC-CFM-CTR-VAN-CMX showed the highest prevalence (12 isolates) in animal feed. On the contrary, the PG-AMC-CFM-CTR-EM-VAN-NIT-CMX pattern revealed the highest prevalence (9 isolates) in food, whereas the PG-AMC-CFM-CTR-VAN-CMX and PG-AMC-CFM-CTR-AZM-EM-TET-LEV-VAN-NIT-CMX patterns revealed the highest prevalence (3 isolates) in diarrhea (Table 6).

**Table 7:** MDR profile of the isolated *Bacillus* spp

Source	Pattern No	Antibiotic Resistance Pattern	No. of antibiotics (classes)	No of resistance isolates	MDR isolates (%)	MAR index	Level of significance
Animal feed	1	CFM	1(1)	1	0 (0)	0.071	NS (*p* = 0.517)
	2	PG-CFM	2 (1)	9	0 (0)	0.142	
	3	PG-AMC-CFM	3(1)	1	0 (0)	0.214	
	4	PG-CFM-TET	3(2)	2	0 (0)	0.214	
	5	PG-CFM-CTR-CMX	4 (1)	2	0 (0)	0.285	
	6	PG-CFM-CTR-NIT	4 (2)	2	0 (0)	0.285	
	7	PG-CFM-TET-EM	4(3)	1	1 (0.6)	0.285	
	8	PG-CFM-CTR-TET-NIT	5 (3)	1	1 (0.6)	0.357	
	9	PG-CFM-CTR-TET-CMX	5 (3)	1	1 (0.6)	0.357	
	10	PG-CFM-CTR-NIT-CMX	5 (3)	3	3 (1.9)	0.357	
	11	PG-AMC-CFM-CTR-VAN-CMX	6 (3)	12	12 (7.7)	0.428	
	12	PG-CTR-AZM-NIT-CMX	5 (4)	1	1 (0.6)	0.357	
	13	PG-CFM-TET-CIP-LEV-VAN	6 (4)	3	3 (1.9)	0.428	
	14	PG-CFM-CTR-EM-VAN-CMX	6 (4)	2	2 (1.2)	0.428	
	15	PG-CFM-CTR-CIP-NIT-CMX	6 (4)	4	4 (2.5)	0.428	
	16	PG-CFM-EM-CIP-LFV-VAN	6 (4)	1	1 (0.6)	0.357	
	17	PG-CFM-LEV-VAN-NIT-CMX	6 (5)	1	1 (0.6)	0.428	
	18	PG-CFM-CIP-VAN-NIT-CMX	6 (5)	2	2 (1.2)	0.428	
	19	CFM-EM-CIP-CL-NIT-CMX	6 (6)	1	1 (0.6)	0.428	
	20	PG-AMC-CFM-CTR-EM-TET-CMX	7 (4)	1	1 (0.6)	0.5	
	21	PG-AMC-CFM-CTR-EM-VAN-CMX	7 (4)	2	2 (1.2)	0.5	
	22	PG-AMC-CFM-CTR-CIP-VAN-CMX	7 (4)	1	1 (0.6)	0.5	
	23	PG-CFM-CIP-LEV-VAN-NIT-CMX	7 (5)	1	1 (0.6)	0.5	
	24	PG-CFM-CTR-EM-VAN-NIT-CMX	7 (5)	1	1 (0.6)	0.5	
	25	PG-CFM-AZM-CIP-LEV-CM-VAN	7 (5)	2	2 (1.2)	0.5	
	26	PG-CFM-CTR-EM-TET-VAN-CMX	7 (5)	2	2 (1.2)	0.5	
	27	PG-CFM-TET-CIP-LEV-VAN-CMX	7 (5)	1	1 (0.6)	0.5	
	28	PG-CFM-CTR-EM-CIP-VAN-CMX	7 (5)	2	2 (1.2)	0.5	
	29	PG-CFM-AZM-EM-CIP-NIT-CMX	7 (5)	1	1 (0.6)	0.5	
	30	PG-CFM-AZM-EM-TET-CM-VAN	7 (5)	1	1 (0.6)	0.5	
	31	PG-CFM-EM-CIP-CM-NIT-CMX	7 (6)	2	2 (1.2)	0.5	
	32	PG-CFM-CTR-CIP-LEV-VAN-NIT-CMX	8 (5)	1	1 (0.6)	0.571	
	33	PG-CFM-CTR-TET-CIP-LEV-VAN-NIT	8 (5)	2	2 (1.2)	0.571	
	34	PG-AMC-CFM-CIP-LEV-CM-NIT-CMX	8 (5)	1	1 (0.6)	0.571	
	35	PG-AMC-CFM-CTR-EM-TET-VAN-CMX	8 (5)	2	2 (1.2)	0.571	
	36	PG-AMC-CFM-CTR-LEV-VAN-NIT-CMX	8 (5)	3	3 (1.9)	0.571	
	37	PG-AMC-CFM-CTR-TET-VAN-NIT-CMX	8 (5)	2	2 (1.2)	0.571	
	38	PG-AMC-CFM-CTR-TET-LEV-VAN-CMX	8 (5)	1	1 (0.6)	0.571	
	39	PG-AMC-CFM-CTR-EM-VAN-NIT-CMX	8 (5)	3	3 (1.9)	0.571	
	40	PG-CFM-AZM-EM-TET-CM-VAN-CMX	8 (6)	2	2 (1.2)	0.571	
	41	PG-AMC-CFM-EM-CIP-CM-NIT-CMX	8 (6)	3	3 (1.9)	0.571	
	42	PG-CFM-TET-CIP-LEV-VAN-NIT-CMX	8 (6)	2	2 (1.2)	0.571	
	43	PG-CFM-CTR-AZM-TET-CIP-NIT-CMX	8 (6)	2	2 (1.2)	0.571	
	44	PG-CFM-CTR-EM-TET-VAN-NIT-CMX	8 (6)	2	2 (1.2)	0.571	
	45	PG-CFM-EM-TET-CIP-VAN-NIT-CMX	8 (7)	1	1 (0.6)	0.571	
	46	PG-AMC-CFM-CTR-AZM-EM-CM-NIT-CMX	9 (5)	2	2 (1.2)	0.642	
	47	PG-AMC-CFM-CTX-AZM-EM-TET-NIT-CMX	9 (5)	1	1 (0.6)	0.642	
	48	PG-AMC-CFM-CTR-TET-CIP-LEV-VAN-CMX	9 (5)	1	1 (0.6)	0.642	
	49	PG-AMC-CFM-CTX-EM-CM-VAN-NIT-CMX	9 (6)	2	2 (1.2)	0.642	
	50	PG-AMC-CFM-CTR-EM-TET-VAN-NIT-CMX	9 (6)	4	4 (2.5)	0.642	
	51	PG-AMC-CFM-CTR-EM-TET-CIP-LEV-VAN-CMX	9 (6)	1	1 (0.6)	0.642	
	52	PG-AMC-CFM-CTR-TET-CIP-VAN-NIT-CMX	9 (6)	1	1 (0.6)	0.642	
	53	PG-AMC-CFM-TET-CIP-LEV-VAN-NIT-CMX	9 (6)	1	1 (0.6)	0.642	
	54	PG-CFM-CTR-EM-TET-LEV-VAN-NIT-CMX	9 (7)	1	1 (0.6)	0.642	
	55	PG-CFM-CTX-EM-TET-CIP-VAN-NIT-CMX	9 (7)	1	1 (0.6)	0.642	
	56	PG-AMC-CFM-CTR-AZM-EM-CM-VAN-NIT-CMX	10 (6)	4	4 (2.5)	0.714	
	57	PG-AMC-CFM-CTR-AZM-ER-TET-CIP-LEV-VAN-CMX	11 (6)	4	4 (2.5)	0.785	
	58	PG-AMC-CFM-CTR-EM-TET-CIP-LEV-VAN-NIT-CMX	11 (7)	5	5 (3.2)	0.785	
	59	PG-AMC-CFM-CTR-AZM-EM-TET-CIP-LEV-CM-VAN-NIT	12 (7)	4	4 (2.5)	0.857	
	60	PG-AMC-CFM-CTR-AZM-EM-TET-CIP-LEV-VAN-NIT-CMX	12 (7)	7	7 (4.5)	0.857	
	61	PG-CFM-CTR-EM-TET-LEV-CM-VAN-NIT-CMX	10 (8)	2	2 (1.2)	0.714	
	62	PG-CFM-CTR-EM-TET-CIP-LEV-CM-VAN-NIT-CMX	11 (8)	1	1 (0.6)	0.785	
	63	PG-AMC-CFM-CTR-EM-TET-CIP-LEV-CM-VAN-NIT-CMX	12 (8)	5	5 (3.2)	0.857	
	64	PG-AMC-CFM-CTR-AZM-EM-TET-CIP-CM-VAN-NIT-CMX	12 (8)	3	3 (1.9)	0.857	
	65	PG-AMC-CFM-CTR-AZM-EM-TET-CIP-LEV-CM-VAN-NIT-CMX	13 (8)	7	7 (4.5)	0.928	
		Total		151	134 (88.7)		
Food	1	PG	1 (1)	1	0 (0)	0.071	
	2	PG-CFM	2 (1)	3	0 (0)	0.142	
	3	PG-AMC-CFM-LEV	4 (2)	1	0 (0)	0.285	
	4	PG-AMC-CFM-LEV-NIT	5 (3)	1	1 (0.6)	0.357	
	5	PG-AMC-CFM-LEV-CMX	5 (3)	2	2 (1.2)	0.357	
	6	PG-CFM-CIP-CM-CMX	5 (4)	1	1 (2.1)	0.357	
	7	PG-CFM-TET-CIP-LEV-VAN	6 (4)	3	3 (6.2)	0.428	
	8	PG-AMC-CFM-CIP-LEV-CM-CMX	8 (4)	1	1 (2.1)	0.571	
	9	CFM-EM-TET-CM-CMX	5 (5)	1	1 (2.1)	0.357	
	10	PG-CFM-EM-TET-CM-CMX	6 (5)	3	3 (6.2)	0.428	
	11	PG-CFM-EM-CIP-CM-CMX	6 (5)	2	2 (4.1)	0.428	
	12	PG-AMC-CFM-CTR-VAN-CMX	6 (3)	1	1 (2.1)	0.428	
	13	PG-AMC-CFM-CTR-VAN-NIT-CMX	7 (4)	2	2 (4.1)	0.5	
	14	PG-AMC-CFM-CTR-CIP-LEV-VAN-CMX	8 (4)	3	3 (6.2)	0.571	
	15	PG-AMC-CFM-CTR-AZM-CM-VAN-CMX	8 (5)	4	4 (8.3)	0.571	
	16	PG-AMC-CFM-CTR-EM-CM-VAN-CMX	8 (5)	1	1 (2.1)	0.571	
	17	PG-AMC-CFM-CTR-EM-VAN-NIT-CMX	8 (5)	9	9 (18.7)	0.571	
	18	PG-AMC-CFM-CTR-AZM-EM-CM-VAN-CMX	9 (5)	3	3 (6.2)	0.642	
	19	PG-AMC-CFM-CTR-EM-TET-VAN-NIT-CMX	9 (6)	5	5 (10.4)	0.642	
	20	PG-AMC-CFM-CTR-AZM-EM-TET-VAN-NIT-CMX	10 (6)	5	5 (10.4)	0.714	
		Total		52	47 (90.3)		
Diarrhea	1	PG-AMC-CFM-CTR-VAN-CMX	6 (3)	3	3 (30.0)	0.428	
	2	PG-AMC-CFM-CTR-AZM-EM-TET-VAN-NIT-CMX	10 (6)	2	2 (20.0)	0.714	
	3	PG-AMC-CFM-CTR-AZM-EM-TET-LEV-VAN-NIT-CMX	11 (7)	3	3 (30.0)	0.785	
	4	PG-AMC-CFM-CTR-AZM-EM-TET-LEV-CM-VAN-NIT-CMX	12 (8)	2	2 (20.0)	0.857	
		Total		10	10 (100)		
Grand Total (overall MDR isolates)				213	191 (89.6)		

*MDR* Multidrug resistance, *PG* Penicillin G, *AMC* Amoxicillin-Clavulanic acid, *CFM* Cefixime, *CTR* Ceftriaxone, *VAN* Vancomycin, *AZM* Azithromycin, *EM* Erythromycin, *TET* Tetracycline, *CM* Clindamycin, *NIT* Nitrofurantoin, *CIP* Ciprofloxacin, *LEV* Levofloxacin, *CMX* Co-Trimoxazole, *NS* Not significant

Furthermore, the MAR index of *Bacillus* spp. was arranged from 0.071–0.928, while *B. cereus* yielded the highest MAR index, ranging from 0.285–0.928, followed by *B. thuringiensis* (0.142–0.857), *B. subtilis* (0.142–0.642), *B. amyloliquefaciens*/*B. licheniformis* (0.142–0.571), *B. megaterium* (0.142–0.285), and *B. coagulans* (0.071–0.142). Regarding sample type, animal feed isolates had the highest MAR index (0.428–0.928), followed by diarrheal isolates (0.428–0.857) and food isolates (0.071–0.714) (Table S10, Table 6). In our study, 90.3% of isolates showed a MAR index > 0.2, with 100, 94.6, and 88.1% from diarrhea, food, and animal feed, respectively. Furthermore, there was a substantial variation (*p* < 0.05) among the MAR index having > 0.2 of seven *Bacillus* strains, while no significant difference (*p* > 0.05) was observed among different sample types (Tables S11 and S12).

## Discussion

In the current study, the isolated *Bacillus* strains were sensitive to GEN, CIP, LEV, CM, AMC, TET, EM, AZM, NIT, and CMX, which was consistent with the previous reports [4, 5, 7, 9, 12, 20, 23, 24]. However, there were species-specific sensitivities of the *Bacillus* strains to CIP, LEV, CM, TET, EM, AZM, and NIT at various doses. Regarding the resistant pattern, diarrheal strains were completely resistant to PG, AMC, CFM, CTR, VAN, and CMX, whereas animal feed-borne strains were generally resistant to PG, AMC, CFM, VAN, and CMX, and food-originated strains were generally resistant to PG, CFM, CTR, and CMX. As for different species, *B*. *cereus*, *B*. *thuringiensis*, and *B*. *licheniformis* strains were all completely resistant to the beta-lactam antibiotics of PG and CFM, while *B*. *subtilis* strains were completely resistant to CFM. Moreover, they were generally resistant to PG, AML, CFM, CTR, VAN, EM, TET, and NIT compared to AZM, CM, CIP, and LEV, which was compatible with the other studies [1, 3, 5, 7, 8, 20, 23–25].

The *B. cereus* strains were generally sensitive to GN, CIP, and LEV, while intermediately sensitive to AZM, CM, and TET. In contrast, they were generally resistant to PG, CFM, CTR, CFM, VAN, EM, AMC, NIT, and TET. This result agreed with earlier findings [4, 7, 20, 23, 24, 26–28], which were isolated from rice, cereals, chicken meat, fresh vegetables, edible fungi, powdered milk, foodstuffs, human stool, and clinical samples. The isolated *B. subtilis* was generally sensitive to GEN, LEV, CM, and CIP; intermediately sensitive to EM, TET, AZM, and NIT; and generally resistant to CFM, PG, CMX, CTR, VAN, and AMC. However, this bacteria strain detected in bread, powdered milk, soil, and shrimp culture ponds showed sensitivity to GEN, VAN, CM, EM, TET, and CMX while being resistant to PG, ampicillin, cefpodoxime, and cefepime [4, 9, 29, 30]. These two dominant species had species-specific responses to AMC, AZM, EM, TET, NIT, and LEV. The main factor might be large abuse, which prolongs time. The high sensitivity to GEN, CIP, and LEV could be attributed to the limited administration of CIP and LEV, while GEN is not absorbed via oral application. Consequently, CTR, CFM, AMC, CMX, and NIT were largely abused in the animal industry and added frequently against infectious diseases. On the other hand, antibiotic regulation contributes to AMR. In several countries, CIP and LEV are prohibited for use on animals due to human drugs.

It is worth mentioning that 89.6% of *Bacillus* isolates were MDR, with 100% of isolates from diarrhea, 90.3% from food, and 88.7% from animal feed exhibiting the MDR pattern. These findings were consistent with other reports that found MDR *Bacillus* spp. in several sources, including food [4, 5, 7, 31, 32]. In our investigation, 100% *B*. *cereus* and 91.8% *B*. *subtilis* isolates yielded MAR indexes  > 0.2, indicating plasmid-mediated resistance and a significant risk of contamination. This implies a high inclination and trend for antibiotic resistance among the MDR bacterial isolates [33]. In Bangladesh, MDRs of *E*. *coli*, *Salmonella* spp., *Campylobacter* spp., and *Enterobacter* spp. were detected in livestock populations due to contamination of animal-derived food and food products [3]. However, MDR in *Bacillus* spp. has not been reported. Our data indicated *Bacillus* strains might produce extended-spectrum beta-lactamase (ESBL) and resistance to third-generation cephalosporins (CFM and CTR), macrolide (EM), tetracycline, second- and third-generation quinolones (CIP and LEV), and sulfonamides (CMX).

In this study, genes encoding β-lactamase (*bla1*, *bla*_TEM_, *bla*_CTX-M-1_, *bla*_CTX-M-2_, *bla*_SHV_), fluroquinolone (*qnr*S), sulfonamide (*sul1*), tetracycline (*tetA*), and macrolide (*erm1*) were derived from animal feed, food, and diarrhea. The most commonly observed β-lactamase genes were *bla1* than *bla*_TEM_, *bla*_CTX-M-1_, *bla*_CTX-M-2_, and *bla*_SHV_ among *Bacillus* strains. Similar detection of the *bla1* gene in *Bacillus* strains was reported by other isolates from chicken meat, meat products, human stool, food, and the environment [27, 34, 35]. However, some of our findings were inconsistent with the previous studies [23, 36, 37]. They observed a higher prevalence of *bla*_TEM_*, bla*_CTX-M-1_, *bla*_CTX-M-2_, and *bla*_SHV_ in waste water, clinical samples, and food samples. The second dominant gene was the *tetA* gene in our study, which was consistent with prior studies [8, 18, 25], but other authors reported a higher occurrence [28, 35]. The prevalence of the erythromycin-resistant gene, *erm1*, was low compared to earlier reports [9, 25, 35, 37]. Furthermore, our study revealed a low prevalence of sulfonamide, *sul1* and quinolone, and *qnrS* resistance genes. The *sul1* gene in *Bacillus* strains isolated from aquaculture ponds was lower than our report [1], whereas the *sul1* gene in wastewater was higher than our report [37]. *Bacillus* spp. are often used as food microbial additives and spread ARGs by horizontal transfer of plasmids, leading to the failure of antibiotic treatment and dramatically altering their phenotypes [4, 5, 38]. The *Bacillus anthrax*-associated plasmids pXO1 and pXO2 have been detected in certain *B*. *cereus* strains with pathogenic potential resembling *B*. *anthracis* [39]. For instance, atypical *B*. *cereus* strains such as *B*. *cereus* G9241, *B*. *cereus* biovar anthracis CA, Bcbva, and Bcbva-like strain BC-AK were linked to anthrax-like disease in mammals, livestock, and humans in the United States, China, and West Africa, implying that it may be widespread [40]. The highly efficient mobilization capacities and horizontal gene transfer may pose a serious threat to gene circulation, particularly ARGs. Probiotic *Bacillus* spp. have already been connected to clinical infections, as well as β-lactams, aminoglycosides, macrolides, chloramphenicol, tetracycline, and erythromycin resistance genes, which may contribute to the spread of ABR in animal microbiota and the possible transmission of ARG to humans [10]. Nevertheless, abusive antibiotic use can transmit antibiotic residues in foods derived from animals, like milk, meat, and eggs, as well as in the environment [6]. As a potential driver of both genes and bacteria resistant to antibiotics, the ARG may be further transmitted to people directly through the food chain [10].

The high resistance of *Bacillus* strains to CFM, CTR, AMC, and PG might be caused by β-lactamase and the presence of ABC (ATP binding cassette) efflux transporters from *B. subtilis* that are tolerant to lincosamide [25, 26]. The different antibiotic mechanisms might be associated with inherent resistance, built-up resistance, gene modification, and DNA transfer that aid in bacterial survival by manipulating the penicillin binding protein (PBP), enzymatic blockage, porin mutations, and efflux pumps [6]. The current study revealed that *Bacillus* isolates primarily carried the β-lactamase resistance genes *bla1*, *bla*_TEM_, *bla*_CTX-M-1_, *bla*_CTX-M-2_, *bla*_SHV_, tetracycline resistance gene *tetA*, and erythromycin resistance gene *erm1*, respectively. It was confirmed that certain ARG classes could be acquired by the majority of antibiotics and evaluated in various *Bacillus* strains. According to resistant gene distribution, 10 distinct ARG patterns were detected in the isolates. The association of the above β-lactamase and other antibiotic genes within the same isolate has been reported [9, 23, 28, 36, 37]. However, the most common associations were *bla1* + *erm1*, *bla1* + *tetA*, *bla1* + *bla*_*TEM*_, *bla*_TEM_ + *tetA*, and *bla*_CTX-M-1_ + *tetA* (Table S2). This occurrence indicates a greater spread of β-lactamase, tetracycline, and erythromycin genes, most likely owing to a genetic component in their mobilization as well as the horizontal transfer of ABR determinants between *Bacillus* strains or from other bacteria into *Bacillus* spp. [7, 8, 36].

In our prior study, we revealed a high *Bacillus* spp. contamination level with significant toxigenic potential in several resources, including animal feed, animal-derived goods, and regular food items [15], where 90.3% of isolates displayed > 0.2 MAR index, indicating a high-risk source of contamination [41]. The presence of MDR and MAR *Bacillus* in animal feed, food, and diarrhea indicated that the abuse of antibiotics poses a severe public health hazard by transmitting AMR to people through the food supply chain.

There is a dearth of research data on the transmission of ARGs and the AMR of *Bacillus* strains in Bangladesh. According to a review report, the emergence of AMR is mainly attributed to antibiotic misuse or overuse by broiler (> 60%) and layer (94.6%) farmers as over-the-counter medication and failure to maintain the drug withdrawal period [3]. Empirical data suggests that antibiotic residues against *Bacillus* spp. exist in the liver and kidney as well as in commercially available feed in Bangladesh, acting as a subtherapeutic dose that hastens the emergence of AMR [15, 42]. Unhygienic livestock and poultry farming in Bangladesh is a significant risk indicator for spreading zoonotic bacteria and antibiotic resistance to people and the environment [3]. Our data confirmed that AMR- *B*. *cereus* strains prevailed in all analyzed samples. The significance of this finding is underlined by earlier research [4, 5, 7, 20, 24, 25] that showed *Bacillus* spp. can transfer ARGs. Bangladesh urgently requires the development of effective surveillance and control plans for the identification and prevention of ABR bacteria utilizing standard antibiotic susceptibility tests in regular animal and human microbiological laboratory settings.

## Conclusion

It is the first investigation of the presence of ARGs of *Bacillus* spp. with public health significance in animal feed, food, and human stool in Bangladesh. The feed- and food-borne *Bacillus* spp. exhibited species-specific trends in both phenotypic and genotypic resistance patterns with respect to antibiotic resistance. The associations of various antibiotic-resistant genes indicated a greater spread of β-lactamase, tetracycline, and erythromycin genes across the food chain. Animal feed and animal-derived products might serve as a channel for *B. cereus* propagation regarding their potential pathogenicity and the development of AMR in humans. This work validates the sources examined as major outlets for the spread of MDR bacteria and ARGs in the food chain of Bangladesh and once again highlights the urgency of a global campaign to combat AMR.

## Materials and methods

### Sampling, selection, isolation, storage, and molecular characterization of *Bacillus *spp. isolates

A total of 218 *Bacillus* spp. isolates were examined and retrieved in our previous study (Table S1), including animal feed (*n* = 90), food (*n* = 40), and human stool (*n* = 50) in southeast Bangladesh [15]. These *Bacillus* spp. strains were initially detected and isolated through cultivation on MYPA (HiMedia, Mumbai, Maharashtra, India) plates, grams staining, biochemical assays, and PCR targeting *16srDNA*, *nheABC*, *hblACD*, *cytK*, and *entFM* genes [15, 43, 44] The strains were preserved in TSB medium (HiMedia, Mumbai, Maharashtra, India) containing 15% glycerol at -80^0^C. The bacterial strains were cultivated aerobically in TSB at 37 °C with agitation at 225 rpm. The Ethical Reviewing Board on Institutional Animal Care and Use Committee at Noakhali Science and Technology University, Bangladesh, granted approval for the experimental protocols. An Informed Consent Form (ICF) was obtained prior to initiating research activities and collecting human stool samples.

### Antibiotic susceptibility test of the *Bacillus* isolates

#### Minimum inhibitory concentration (MIC)

To assess any link between antimicrobial resistance patterns and the chosen antibiotic category, the antibiogram profile of isolates was tested by estimating the MIC in appropriate broth using sterile U bottom 96-well plates with lids (SPL Life Science, Pochon, Kyonggi-do, South Korea) employing the microtiter broth dilution method [45]. The MIC represented the lowest level of antimicrobial that completely inhibited the growth of the organism. According to 2020 Clinical and Laboratory Standards Institute (CLSI) criteria, susceptible, intermediate, and resistant MIC (μg/ml) interpretation was done ([45], Table S13). Briefly, 2–3 single pure fresh colonies of *Bacillus* spp. grown on NA (24 h old) were inoculated into 5 ml of MHB (HiMedia, Mumbai, Maharashtra, India) and kept at 37^0^C for up to 8 h. To standardize the turbidity of bacterial suspension, MHB was used to achieve a turbidity of 0.5 McFarland concentration (1 × 10^5^ CFU/mL) through a two-fold serial dilution of antibiotics through visual evaluation with a card featuring a white backdrop and distinct black lines. The microtiter plates were incubated in a shaking incubator at 37^0^C. The lowest concentration that inhibited indicator strains growth was noted. All MIC tests were done in triplicate. *Staphylococcus aureus* ATCC 29213 was used as a positive control.

#### Minimum Bactericidal Concentration (MBC)

Five microliters of inoculum from the MIC experiment's well that had no bacterial growth after 24 h were spotted on NA (HiMedia, Mumbai, Maharashtra, India). The plates were incubated at 37^0^C for 16 to 24 h in order to evaluate the MBC described earlier [46]. MBC was set as the lowest level on a NA plate where there was no visual growth. *Bacillus* spp. was detected, and it was determined that the growth of bacteria was bacteriostatic, while the absence of growth indicated bactericidal effects. The NA plate was cultivated with the indicated inoculum dilution to test for contamination and cell viability [45]. All analyses were performed in triplicate.

### Determination of multidrug resistant (MDR) and multiple antibiotic resistance (MAR) index

MDR was considered to have at least one agent that was resistant to three or more types of antibiotics [47]. The following 14 antibiotics procured in powdered form from Sisco Research Laboratories Pvt. Ltd. (SRL, E, Mumbai, Maharashtra 400,099, India) were used: Penicillin G, PG (0.25–32 µg/mL); Amoxicillin + Clavulanic acid, AMC (0.01–0.5 µg/mL); Cefixime, CFM (0.5–4 µg/mL); Ceftriaxone, CTR (1–8 µg/mL); Azithromycin, AZM (0.5–8 µg/mL); Erythromycin, EM (0.25–32 µg/mL); Tetracycline, TET (0.25–32 µg/mL); Ciprofloxacin, CIP (0.12–16 µg/mL); Levofloxacin, LEV (0.12–16 µg/mL); Clindamycin, CM (0.25–8 µg/mL); Vancomycin, VAN (0.5–32 µg/mL); Gentamicin, GEN (0.5–32 µg/mL); Nitrofurantoin, NIT (32–128 µg/mL), and Co-Trimoxazole, CMX (1–128 µg/mL). The MAR index of isolated *Bacillus* spp. was determined as a/b, where “a” is the number of antibiotics to which a strain is resistant and “b” is the total number tested [41].

### Resistance genotyping

The genomic DNA of various *Bacillus* species isolated from animal feed, food, and stool samples was extracted utilizing the TaKaRa MiniBEST Bacteria Genomic DNA Extraction Kit Ver.3.0 (GW Vitek, Seoul, Korea). Following the manufacturer's instructions, pure colonies of *Bacillus* spp. isolates were prepared for DNA extraction from Tryptic Soy Broth (TSB) and Luria Bertani (LB) broth. A NanoDropTM 8000 spectrophotometer (Thermo Scientific, California, USA) was utilized to measure the concentration and purity of the eluted DNA.

Phenotypically resistant *Bacillus* spp. were screened by PCR for 9 ARGs, including 5 β-lactamase (*bla1*, *bla*_TEM_, *bla*_CTX-M-1_, *bla*_CTX-M-2_, and *bla*_SHV_), single fluroquinolone (*qnr*S), sulfonamide (*sul1*), tetracycline (*tetA*), and macrolide (*erm1*). PCR protocols were followed exactly as reported earlier [1, 8, 23, 36, 48–50] (Table 8, Table S14). *B. cereus* ATCC 14579 and *E. coli* ATCC 25922 served as positive controls, while sterile Milli-Q water (Sigma Aldrich, Bengaluru, Karnataka 560,099, India) served as a negative control. The PCR reaction was performed in a 25 µl volume with OneTaqQuick-Load 2 × Master Mix (New England Biolabs Inc., United States), 0.2 µmol L^−1^ final concentration of each primer, and 2.5 µl of ready DNA template. PCR was conducted on a T100 Thermal cycler (Bio-Rad, United States). The PCR products were analyzed on 1.5% agarose gel (MP Biomedicals LLC, United States) with a Mini-Sub Cell GT Horizontal Electrophoresis System (Bio-Rad, United States), stained with Ethidium bromide (EtBr), displayed with a UV transilluminator (Gel Doc EZ), and imaged using a gel documentation system.

**Table 8:** Primer used in this study

Primer	Sequence (5´ → 3´)	Annealing temperature (^o^C)	Product size (bp)	Reference
*bla1*	F = CATTGCAAGTTGAAGCGAAA	50	680	[27]
	R = TGTCCCGTAACTTCCAGCTC			
*bla*_*TEM*_	F = ATGAGTATTCAACATTTCCG	55	850	[25]
	R = CCAATGCTTAATCAGTGAGG			
*bla*_CTX-M-1_	F = AAAAATCACTGCGCCAGTTC	52	415	[25]
	R = AGCTTATTCATCGCCACGTT			
*bla*_CTX-M-2_	F = CGACGCTACCCCTGCTATT	52	552	[25]
	R = CCAGCGTCAGATTTTTCAGG			
*bla*_*S*HV_	F = GCGAAAGCCAGCTGTCGGGC	62	304	[26]
	R = GATTGGCGGCGCTGTTATCGC			
*qnr*S	F = GCAAGTTCATTGAACAGGGT	54	428	[28]
	R-TCTAAACCGTCGAGTTCGGCG			
*sul1*	F = CGGCGTGGGCTACCTGAACG	57	433	[1]
	R = GCCGATCGCGTGAAGTTCCG			
*tetA*	F = GGCGGTCTTCTTCATCATGC	58	502	[8]
	R = CGGCAGGCAGAGCAAGTAGA			
*ermA*	F = TCTAAAAAGCATGTAAAAGAA	52	645	[50]
	R = CTTCGATAGTTTATTAATATTAGT			

### Statistical analysis

The antibiotic susceptibility results were provided in MS-2016 Excel sheets and analyzed using IBM SPSS Statistics version 24 (SPSS Inc., Chicago, IL, USA). The prevalence was computed by a descriptive study and the Chi-square test, and the degree of significance was established using Pearson correlation coefficients. The statistical significance was calculated as ^∗^*p* < 0.05 and ^∗∗^*p* < 0.01.

### Supplementary Information

**Additional file 1: Figure S1.** Cultural morphology and Grams staining  of isolated *Bacillus* spp.** Figure S2.***16srDNA* gene of the isolated *Bacillus* spp. by PCR test. **Figure S3.** Toxin genes (*nheA*, *nheB*, *nheC*, *cytK*) of isolated *Bacillus* spp. by PCR test. **Figure S4.** Toxin genes (*hblA*, *hblC*, *hblD*, *entFM*) of isolated *Bacillus* spp. by PCR test. **Figure S5.** Antibiotic resistance genes of isolated *Bacillus* spp. by PCR test.** Table S1.** Strains and source of selected *Bacillus* spp.** Table S2.** Biochemical characteristics of *Bacillus* spp.** Table S3.** Primers of toxin gene and *16srRNA* gene and PCR protocol used in this study. **Table S4.** Overall antimicrobial susceptibility profile of *Bacillus* spp.** Table S5.** Overall antimicrobial susceptibility profiles of 7 *Bacillus* species. **Table S6.** Distributions of antibiotic genes among animal feed, food and diarrhea. **Table S7.** Prevalence of ARGs of *Bacillus* spp. in animal feed, food and diarrhea. **Table S8.** Prevalence of ARGs of 7 *Bacillus* species. **Table S9.** Distributions of ARGs among 7 *Bacillus* species. **Table S10.** MDR profiles of *B*. *cereus*, *B*. *subtilis*, *B*. *amyloliquefaciens*, *B*. *licheniformis*, *B*. *thuringiensis*, *B*. *megaterium* and *B*. *coagulans*. **Table S11.** Species wise percentage of MAR index >0.2. **Table S12.** Sample wise percentage of MAR index >0.2. **Table S13.** MIC break point of Antibiotic used. **Table S14.** PCR protocol of antibiotic resistant gene primer used in this study.

## Data Availability

The dataset can be accessed upon a reasonable request made to the Corresponding author.

## Abbreviations

AMR: Antimicrobial resistance
ARG: Antibiotic resistant gene
CDC: Center for Disease Control and Prevention
CFU: Colony-forming unit
CLSI: Clinical and Laboratory Standards Institute
DNA: Deoxyribonucleic acid
EtBr: Ethidium bromide
LB: Luria-Bertani
MAR: Multiple antibiotic resistance
MBC: Minimum bactericidal concentration
MDR: Multidrug-resistant
MHB: Muller Hinton Broth
MIC: Minimum inhibitory concentration
MYPA: Mannitol-Egg-Yolk-Polymyxin-Agar
NA: Nutrient Agar
PCR: Polymerase chain reaction
TSB: Tryptic soy broth
WHO: World Health Organization
