# Supplementary material for: Emergence of multidrug-resistant Bacillus spp. derived from animal feed, food and human diarrhea in South-Eastern Bangladesh
Source: BMC Microbiol. 2024 Feb 19;24:61. doi: 10.1186/s12866-024-03199-3 (PMC10875756; doi:10.1186/s12866-024-03199-3)
Supplement: Supplementary file 1 — Additional file 1: Figure S1. Cultural morphology and Grams staining of isolated Bacillus spp. Figure S2.16srDNA gene of the isolated Bacillus spp. by PCR test. Figure S3. Toxin genes (nheA, nheB, nheC, cytK) of isolated Bacillus spp. by PCR test. Figure S4. Toxin genes (hblA, hblC, hblD, entFM) of isolated Bacillus spp. by PCR test. Figure S5. Antibiotic resistance genes of isolated Bacillus spp. by PCR test. Table S1. Strains and source of selected Bacillus spp. Table S2. Biochemical characteristics of Bacillus spp. Table S3. Primers of toxin gene and 16srRNA gene and PCR protocol used in this study. Table S4. Overall antimicrobial susceptibility profile of Bacillus spp. Table S5. Overall antimicrobial susceptibility profiles of 7 Bacillus species. Table S6. Distributions of antibiotic genes among animal feed, food and diarrhea. Table S7. Prevalence of ARGs of Bacillus spp. in animal feed, food and diarrhea. Table S8. Prevalence of ARGs of 7 Bacillus species. Table S9. Distributions of ARGs among 7 Bacillus species. Table S10. MDR profiles of B. cereus, B. subtilis, B. amyloliquefaciens, B. licheniformis, B. thuringiensis, B. megaterium and B. coagulans. Table S11. Species wise percentage of MAR index >0.2. Table S12. Sample wise percentage of MAR index >0.2. Table S13. MIC break point of Antibiotic used. Table S14. PCR protocol of antibiotic resistant gene primer used in this study. [file 12866_2024_3199_MOESM1_ESM.docx]

**Emergence of multidrug-resistant *Bacillus* spp. derived from animal feed, food and human diarrheal cases in South-eastern Bangladesh**

**Md Atiqul Haque^1,2#^, Huilong Hu^1#^,** **Jiaqi Liu^1^, Md Aminul Islam^3^, Foysal Hossen^3^, Md Arifur Rahman^3^, Firoz Ahmed^3*^, and Cheng He^1*^**

^1^Key Laboratory of Animal Epidemiology and Zoonosis, Ministry of Agriculture, College of Veterinary Medicine, China Agricultural University, Beijing, 100019, China

^2^Department of Microbiology, Faculty of Veterinary and Animal Science, Hajee Mohammad Danesh Science and Technology University, Dinajpur-5200, Bangladesh

^3^Department of Microbiology, Faculty of Science, Noakhali Science and Technology University, Noakhali-3814, Bangladesh

# Contribute to the work equally

^*^ Corresponding author: [hecheng@cau.edu.cn](mailto:hecheng@cau.edu.cn); [firoz@nstu.edu.bd](mailto:firoz@nstu.edu.bd)

**List of Index**

| S/N | Additional file | Description | Page no. |
| --- | --- | --- | --- |
| 1 | Additional file 1 | Figure S1. Cultural morphology and Grams staining of isolated *Bacillus* spp. | 3 |
| 2 | Additional file 2 | Figure S2. *16srDNA* gene of the isolated *Bacillus* spp. by PCR test | 4 |
| 3 | Additional file 3 | Figure S3. Toxin genes (*nheA*, *nheB*, *nheC*, *cytK*) of isolated *Bacillus* spp. by PCR test | 5 |
| 4 | Additional file 4 | Figure S4. Toxin genes (*hblA*, *hblC*, *hblD*, *entFM*) of isolated *Bacillus* spp. by PCR test | 6 |
| 5 | Additional file 5 | Figure S5. Antibiotic resistance genes of isolated *Bacillus* spp. by PCR test. | 7 |
| 6 | Additional file 6 | Table S1. Strains and source of selected *Bacillus* spp. | 8 |
| 7 | Additional file 7 | Table S2. Biochemical characteristics of *Bacillus* spp. | 9 |
| 8 | Additional file 8 | Table S3. Primers of toxin gene and *16srRNA* gene and PCR protocol used in this study | 10 |
| 9 | Additional file 9 | Table S4. Overall antimicrobial susceptibility profile of *Bacillus* spp. | 10 |
| 10 | Additional file 10 | Table S5. Overall antimicrobial susceptibility profiles of 7 *Bacillus* species | 11 |
| 11 | Additional file 11 | Table S6. Distributions of antibiotic genes among animal feed, food and diarrhea | 12 |
| 12 | Additional file 12 | Table S7. Prevalence of ARGs of *Bacillus* spp. in animal feed, food and diarrhea | 13 |
| 13 | Additional file 13 | Table S8. Prevalence of ARGs of 7 *Bacillus* species | 13 |
| 14 | Additional file 14 | Table S9. Distributions of ARGs among 7 *Bacillus* species | 14-15 |
| 15 | Additional file 15 | Table S10. MDR profiles of *B*. *cereus*, *B*. *subtilis*, *B*. *amyloliquefaciens*, *B*. *licheniformis*, *B*. *thuringiensis*, *B*. *megaterium* and *B*. *coagulans* | 16-18 |
| 16 | Additional file 16 | Table S11. Species wise percentage of MAR index >0.2 | 19 |
| 17 | Additional file 17 | Table S12. Sample wise percentage of MAR index >0.2 | 19 |
| 18 | Additional file 18 | Table S13. MIC break point of Antibiotic used | 20 |
| 19 | Additional file 19 | Table S14. PCR protocol of antibiotic resistant gene primer used in this study | 20 |


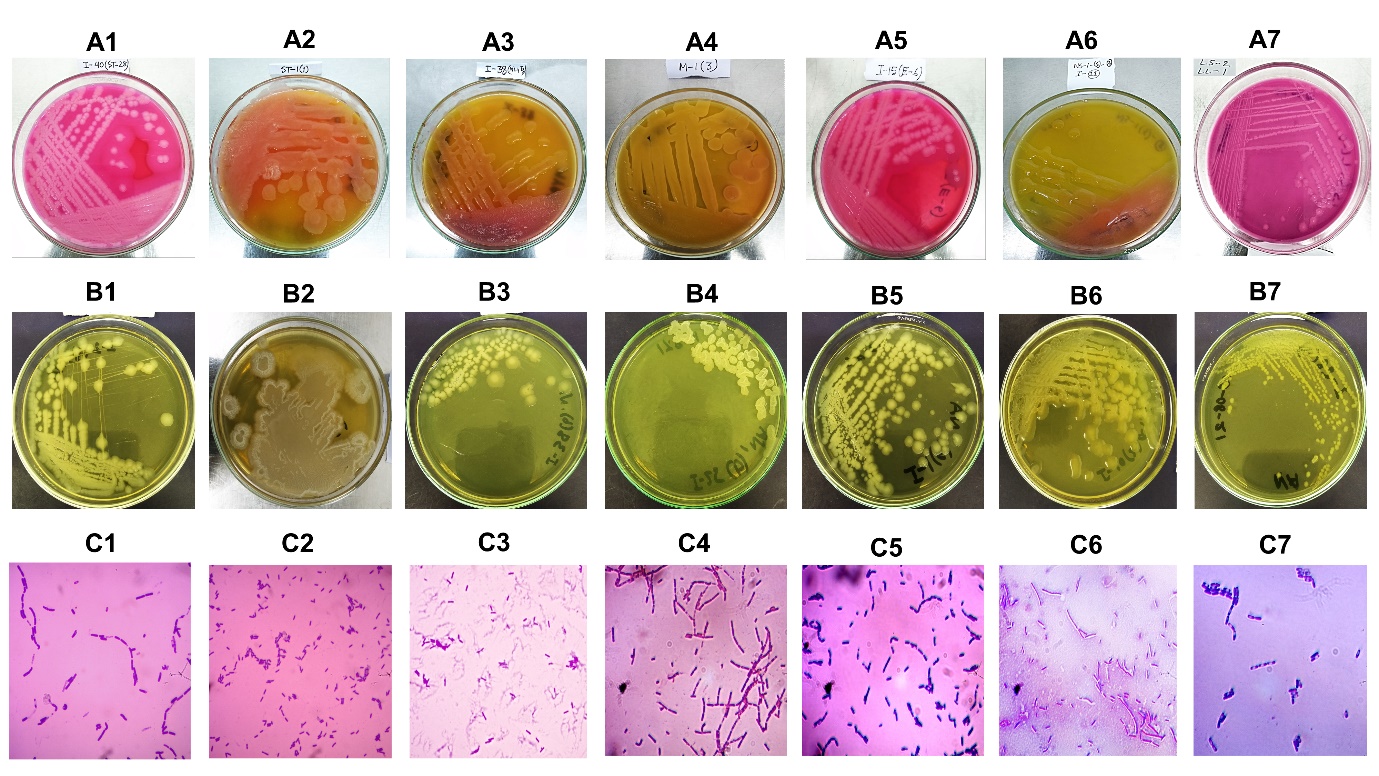


**Figure S1:** Cultural morphology and Grams staining of isolated *Bacillus* spp. (A1) *B*. *cereus* on MYPA; (A2) *B*. *subtilis* on MYPA; (A3) *B*. *amloliquefaciens* on MYPA; (A4) *B*. *licheniformis* on MYPA; (A5) *B*. *thuringiensis* on MYPA; (A6) *B*. *megaterium* on MYPA; (A7) *B*. *coagulans* on MYPA; (B1) *B*. *cereus* on NA; (B2) *B*. *subtilis* on NA; (B3) *B*. *amloliquefaciens* on NA; (B4) *B*. *licheniformis* on NA; (B5) *B*. *thuringiensis* on NA; (B6) *B*. *megaterium* on NA; (B7) *B*. *coagulans* on NA; (C1) *B*. *cereus* on Gram stain (100X); (B2) *B*. *subtilis* on Gram stain (100X); (B3) *B*. *amloliquefaciens* on Gram stain (100X); (B4) *B*. *licheniformis* on Gram stain (100X); (B5) *B*. *thuringiensis* on Gram stain (100X); (B6) *B*. *megaterium* on Gram stain (100X); (B7) *B*. *coagulans* on Gram stain (100X).


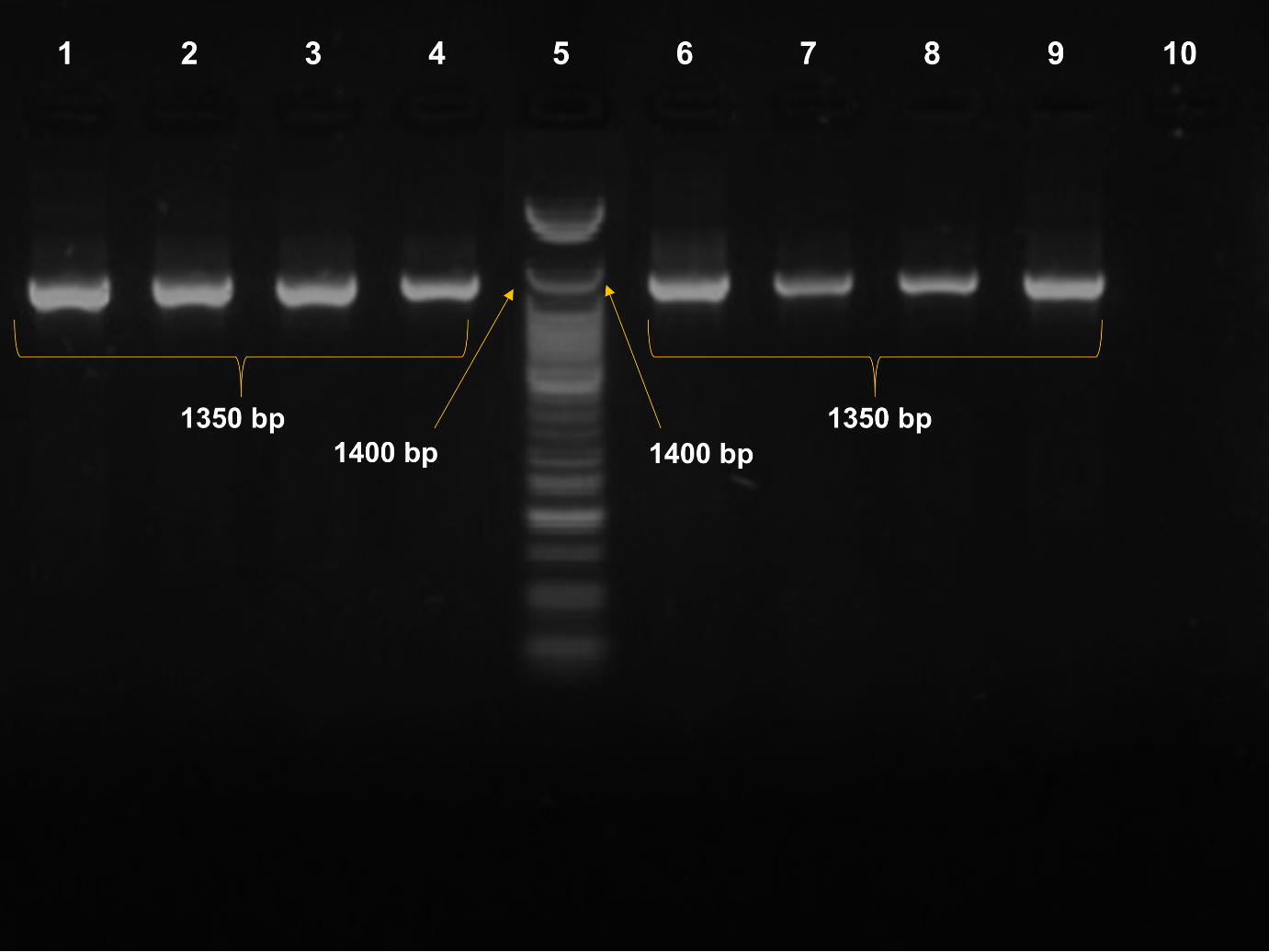


**Figure S2:** *16srDNA* gene of the isolated *Bacillus* spp. by PCR test. L1: *B*. *cereus*, L2: *B*. *subtilis*, L3: *B*. *amyloliquefaciens*, L4: *B*. *licheniformis*, L5: 100bp DNA ladder, L6: *B*. *thuringiensis*, L7: *B*. *megaterium*, L8: *B*. *coagulans*, L9: positive control L10: negative control.


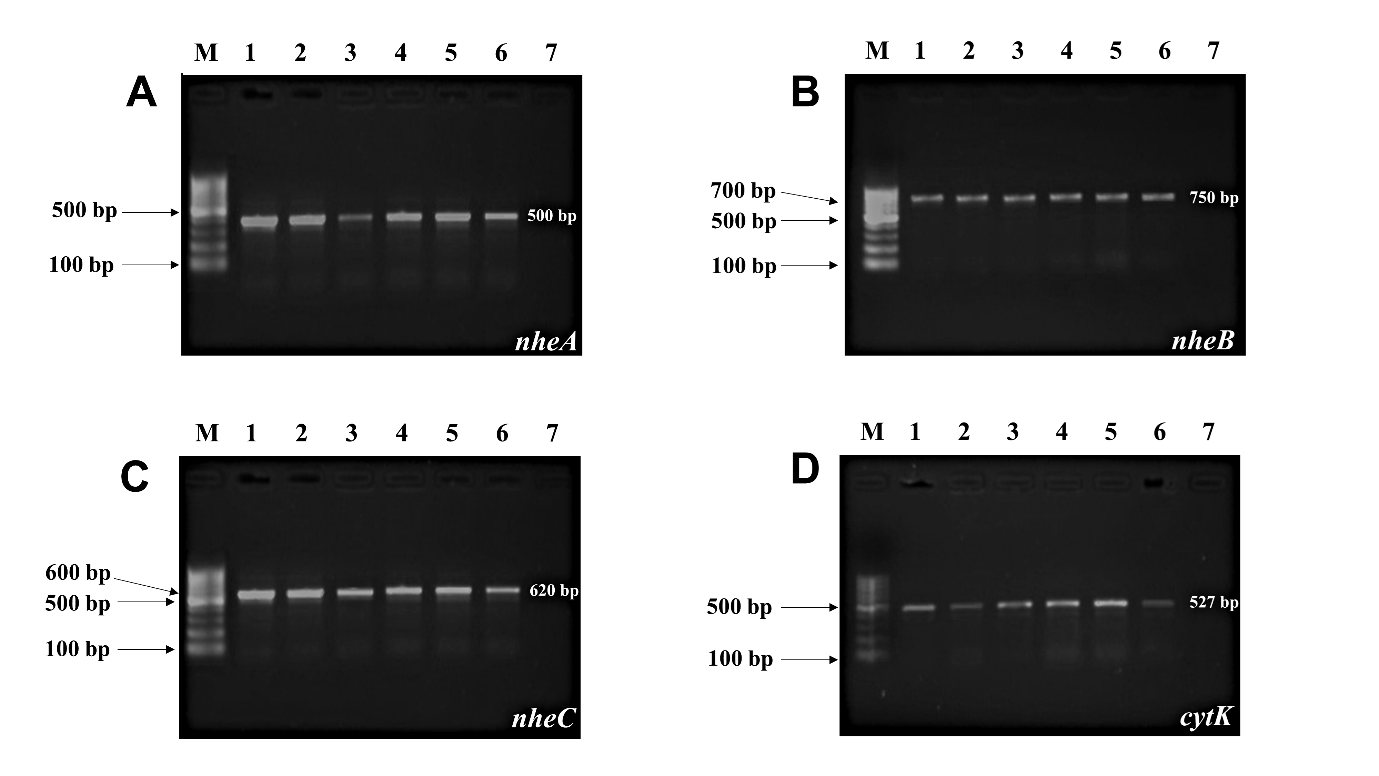


**Figure S3:** Toxin genes (*nheA*, *nheB*, *nheC*, *cytK*) of isolated *Bacillus* spp. by PCR test. (A) *nheA* gene, M: 100 bp marker, L1: positive control, L2: sample 1, L3: sample 2, L4: sample 3, L5: sample 4, L6: sample 5, L7: negative control; (B) *nheB* gene, M: 100 bp marker, L1: positive control, L2: sample 1, L3: sample 2, L4: sample 3, L5: sample 4, L6: sample 5, L7: negative control; (C) *nheC* gene, M: 100 bp marker, L1: positive control, L2: sample 1, L3: sample 2, L4: sample 3, L5: sample 4, L6: sample 5, L7: negative control; (D) *cytK* gene, M: 100 bp marker, L1: positive control, L2: sample 1, L3: sample 2, L4: sample 3, L5: sample 4, L6: sample 5, L7: negative control.


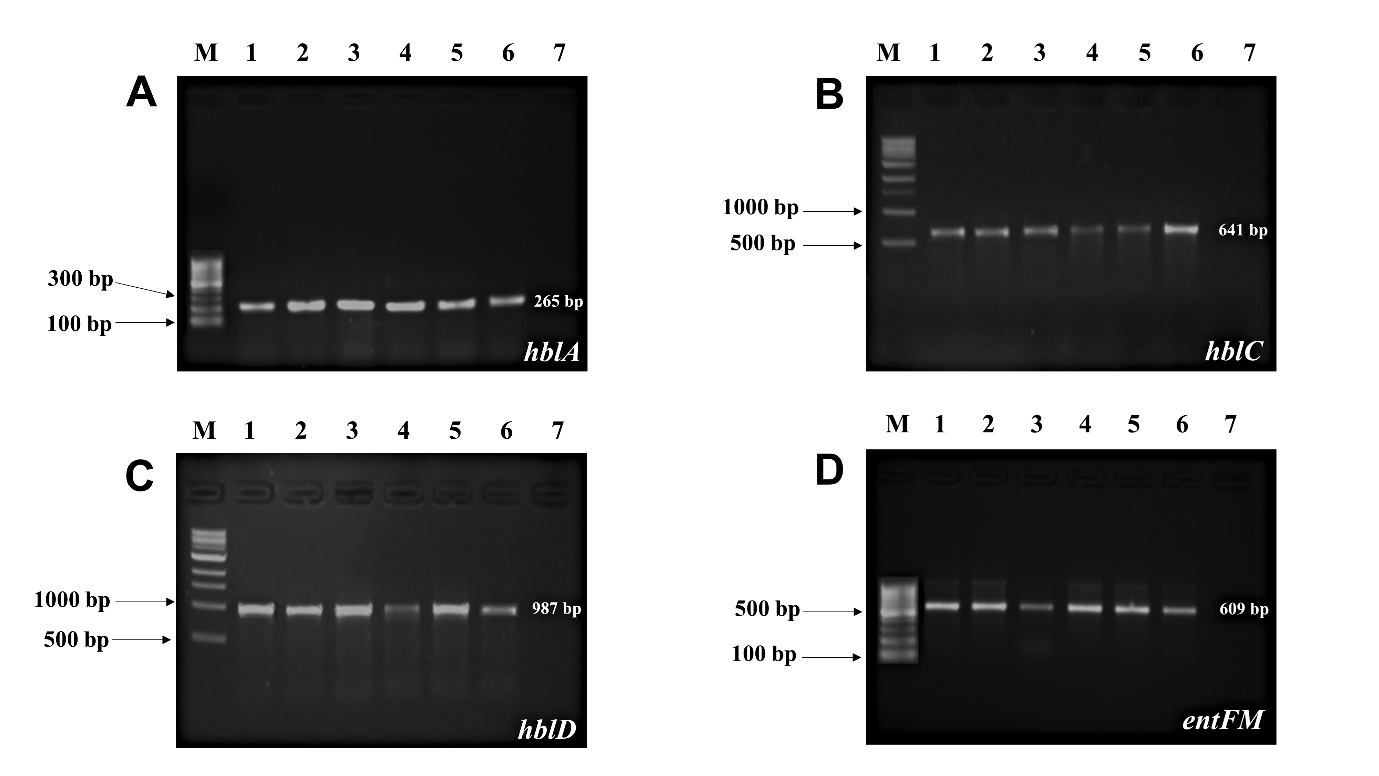


**Figure S4:** Toxin genes (*hblA*, *hblC*, *hblD*, *entFM*) of isolated *Bacillus* spp. by PCR test. (A) *hblA* gene, M: 100 bp marker, L1: positive control, L2: sample 1, L3: sample 2, L4:sample 3, L5: sample 4, L6: sample 5, L7: negative control; (B) *hblC* gene, M: 1 kb marker, L1: positive control, L2: sample 1, L3: sample 2, L4: sample 3, L5:sample 4, L6: sample 5, L7: negative control; (C) *hblD* gene, M: 1 kb marker, L1: positive control, L2: sample 1, L3: sample 2, L4: sample 3, L5: sample 4, L6:sample 5, L7: negative control; (D) *entFM* gene, M: 100 bp marker, L1: positive control, L2: sample 1, L3: sample 2, L4: sample 3, L5: sample 4, L6: sample 5, L7: negative control.


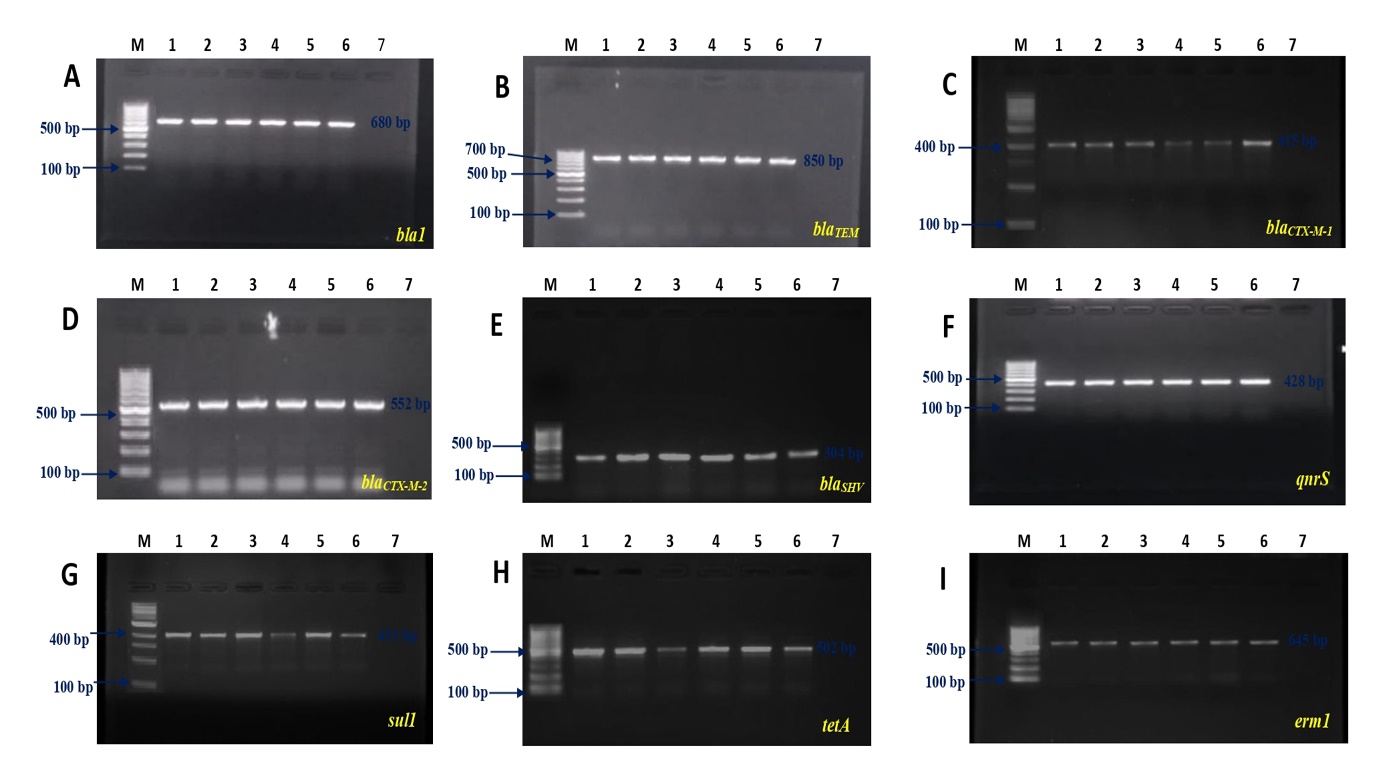


**Fig. S5. Antibiotic resistance genes of isolated *Bacillus* spp. by PCR test.** **(****A)** (*bla1* gene, L1: 100 bp marker, L2: positive control, L3: sample 1, L4: sample 2, L5: sample 3,L6: sample 4, L7: sample 5, L8: negative control); **(B)** (*bla*_TEM_ gene, L1: 100 bp marker, L2: positive control, L3: sample 1, L4: sample 2, L5: sample 3, L6: sample 4,L7: sample 5, L8: negative control); **(C)** (*bla*_CTX-M-1_ gene, L1: 100 bp marker, L2: positive control, L3: sample 1, L4: sample 2, L5: sample 3, L6: sample 4, L7: sample 5,L8: negative control); **(D)** (*bla*_CTX-M-2_ gene, L1: 100 bp marker, L2: positive control, L3: sample 1, L4: sample 2, L5: sample 3, L6: sample 4, L7: sample 5, L8: negative control); **(E)** (*bla*_SHV_ gene, L1: 100 bp marker, L2: positive control, L3: sample 1, L4: sample 2, L5: sample 3,L6: sample 4, L7: sample 5, L8: negative control); **(F)** (*qnrS* gene, L1: 100 bp marker, L2: positive control, L3: sample 1, L4: sample 2, L5: sample 3, L6: sample 4,L7: sample 5, L8: negative control); **(G)** (*sul1* gene, L1: 100 bp marker, L2: positive control, L3: sample 1, L4: sample 2, L5: sample 3, L6: sample 4, L7: sample 5,L8: negative control); **(H)** (*tetA* gene, L1: 100 bp marker, L2: positive control, L3: sample 1, L4: sample 2, L5: sample 3, L6: sample 4, L7: sample 5, L8: negative control); **(I)** (*erm1*  gene, L1: 100 bp marker, L2: positive control, L3: sample 1, L4: sample 2, L5: sample 3, L6: sample 4, L7: sample 5, L8: negative control).

**Table S1. Strains and source of selected *Bacillus* spp.**

| Sample type | | Isolated bacteria | Positive species | | | | | | |
| --- | --- | --- | --- | --- | --- | --- | --- | --- | --- |
|  |  |  | *B. cereus* | *B. subtilis* | *B. amylolequefaciens* | *B. licheniformis* | *B. thuringiensis* | *B. megaterium* | *B. coagulans* |
| Animal feed (*n*=90) | LF (*n*=20) | 42 | 22 (52.3) | 12 (28.5) | 1 (2.3) | 3 (7.1) | 2 (4.7) | 2 (4.7) | 0 (0.0) |
|  | BF (*n*=20) | 37 | 19 (51.3) | 9 (24.3) | 2 (5.4) | 2 (5.4) | 3 (8.1) | 1 (2.7) | 1 (2.7) |
|  | DF (*n*=15) | 26 | 14 (53.8) | 5 (19.2) | 4 (15.3) | 1 (3.8) | 1 (3.8) | 1 (3.8) | 0 (0.0) |
|  | CF (*n*=20) | 28 | 12 (42.8) | 7 (25.0) | 0 (0.0) | 5 (17.8) | 0 (0.0) | 3 (10.7) | 1 (3.5) |
|  | FF (*n*=15) | 19 | 8 (42.1) | 5 (26.3) | 1 (5.2) | 2 (10.5) | 0 (0.0) | 1 (5.2) | 2 (10.5) |
|  | Total | 152 | 75 (49.3) | 38 (25.0) | 8 (5.2) | 13 (8.5) | 6 (3.9) | 8 (5.2) | 4 (2.6) |
| Food  (*n*=40) | E (*n*=20) | 25 | 14 (56.0) | 0 (0.0) | 8 (32.0) | 0 (0.0) | 3 (12.0) | 0 (0.0) | 0 (0.0) |
|  | M (*n*=20) | 31 | 16 (51.6) | 8 (25.8) | 4 (12.9) | 0 (0.0) | 2 (6.4) | 0 (0.0) | 1 (3.2) |
|  | Total | 56 | 30 (53.5) | 8 (14.2) | 12 (21.4) | 0 (0.0) | 5 (8.9) | 0 (0.0) | 1 (1.7) |
| Diarrhea  (*n*=50) | HS (*n*=50) | 10 | 7 (70.0) | 3 (30.0) | 0 (0.0) | 0 (0.0) | 0 (0.0) | 0 (0.0) | 0 (0.0) |
|  | Total | 10 | 7 (70.0) | 3 (30.0) | 0 (0.0) | 0 (0.0) | 0 (0.0) | 0 (0.0) | 0 (0.0) |

LF=Layer feed, BF=Broiler feed, DF=Duck feed, CF=Cattle feed, FF=Fish feed, E=Egg, M=Milk, HS=Human stool

**Supplementary Table S2. Biochemical characteristics of *Bacillus* spp.**

| Test | Bacterial species | | | | | | |
| --- | --- | --- | --- | --- | --- | --- | --- |
|  | *B. cereus* | *B. thuringiensis* | *B. amyloliquefaciens* | *B. subtilis* | *B. licheniformis* | *B. megaterium* | *B. coagulans* |
| Gram stain | + | + | + | + | + | + | + |
| Catalase | + | + | + | + | + | + | + |
| Egg yolk reaction | + | + | - | - | + | - | - |
| Motility | + | + | + | - | - | - | - |
| Mannitol | - | - | + | + | + | + | - |
| Citrate | + | + | + | + | + | + | - |
| VP | + | + | + | + | + | - | - |
| Nitrate reduction | + | + | + | + | + | - | - |
| Indole | - | - | - | - | - | - | - |
| Starch hydrolysis | + | + | + | + | + | + | + |
| Acid from |  |  |  |  |  |  |  |
| Glucose | + | + | + | + | + | + | + |
| Lactose | - | - | + | + | + | + | + |
| Sucrose | - | - | + | + | + | + | + |
| Oxidase | - | - | + | + | - | - | - |
| Anaerobic growth | + | + | - | - | + | - | + |

**Supplementary Table S3. Primers of toxin gene and *16srRNA* gene and PCR protocol used in this study**

| Name | Sequence | Product size (bp) | PCR condition | Reference |
| --- | --- | --- | --- | --- |
| *nheA* | F=TTTCTATCGGTACTTTAAGTAATGAAATTGTA | 405 | 95°C for 15 min; 35 cycles of 94°C for 45 sec, 63.7°C for 1 min, and 72°C for 10 min; elongation step at 72°C for 10 min | Sergeev et al., 2006 |
|  | R=AACTGTTTAATGTACTTCAACGTTTGTAAC |  |  |  |
| *nheB* | F=TTATAAAGTAATGGCTCTATCAGCACT | 750 | 95°C for 15 min; 35 cycles of 94°C for 45 sec, 62.1°C for 1 min, and 72°C for 10 min; elongation step at 72°C for 10 min |  |
|  | R=TACTGCACCACCGATAATTGCAA |  |  |  |
| *nheC* | F=GTTCAGTTGTGAGCAGGAGCTT | 620 | 95°C for 15 min; 35 cycles of 94°C for 45 sec, 60.7°C for 1 min, and 72°C for 10 min; elongation step at 72 °C for 10 min |  |
|  | R=AAACTATTTGTATCTTTCGCCATTCTAT |  |  |  |
| *CytK* | F=GTAACAGATATCGGKCAAAATGCA | 527 | 95°C for 15 min; 35 cycles of 94°C for 45 sec, 60.7°C for 1 min, and 72°C for 10 min; elongation step at 72 °C for 10 min |  |
|  | R=TGTTATATCCRTTAAAGAATACGTTCCA |  |  |  |
| *hblA* | F=CGACGCTATTAACTATTACAACTGCTA | 265 | 95°C for 15 min; 35 cycles of 94°C for 45 sec, 62.1°C for 1 min, and 72°C for 10 min; elongation step at 72°C for 10 min |  |
|  | R=GTAACAGCATGTGCCCTTGCA |  |  |  |
| *hblC* | F=TATAACAAAGGAAAAGAAATTAACAACTCTA | 641 | 95°C for 15 min; 35 cycles of 94°C for 45 sec, 62.1°C for 1 min, and 72°C for 10 min; elongation step at 72°C for 10 min |  |
|  | R=CATGACTATTCTCCTTCTTTCGCTAA |  |  |  |
| *hbld* | F=TGCACAAGAAACGACCGCTCA | 987 | 95°C for 15 min; 35 cycles of 94°C for 45 sec, 62.1°C for 1 min, and 72°C for 10 min; elongation step at 72°C for 10 min |  |
|  | R=ATAATTTGCGCCCATTGTATTCCAT |  |  |  |
| *entFM* | F=AAAGAAATTAATGGACAAACTCAAACTCA | 609 | 95°C for 15 min; 35 cycles of 94°C for 45 sec, 63°C for 1 min, and 72°C for 10 min; elongation step at 72°C for 10 min |  |
|  | R=GTATGTAGCTGGGCCTGTACGT |  |  |  |
| *16srRNA* | F=AGAGTTTGATCCTGGCTCAG | 1350 | 94°C for 3 min; 35 cycles of 94°C for 45 sec, 56°C for 45 sec, and 72°C for 45 sec; elongation step at 72°C for 7 min | Saeed et al., 2020 |
|  | R=GGTTACCTTGTTACGACTT |  |  |  |

**Table S4. Overall antimicrobial susceptibility profile of *Bacillus* spp. (*n*=218)**

| Antibiotic | MIC range (µg/ml) | S (%) | I (%) | R (%) |
| --- | --- | --- | --- | --- |
| PG | 0.25-32 | 9 (4.1) | 0 (0) | 209 (95.8) |
| AMC | 0.01-0.5 | 82 (37.6) | 0 (0) | 136 (62.3) |
| CFM | 0.5-4 | 6 (2.7) | 0 (0) | 212 (97.2) |
| CTR | 1-8 | 18 (8.2) | 41 (18.8) | 159 (72.9) |
| VAN | 0.5-32 | 12 (5.5) | 50 (22.9) | 156 (71.5) |
| AZM | 0.5-8 | 73 (33.4) | 86 (39.4) | 59 (27.06) |
| EM | 0.25-32 | 41 (18.8) | 55 (25.2) | 122 (55.9) |
| TET | 0.25-32 | 56 (25.6) | 59 (27.1) | 103 (47.2) |
| GEN | 0.5-32 | 218 (100) | 0 (0) | 0 (0) |
| CM | 0.25-8 | 114 (52.2) | 45 (20.6) | 59 (27.1) |
| NIT | 32-128 | 46 (21.1) | 51 (23.3) | 121(55.5) |
| CIP | 0.12-16 | 136 (62.3) | 0 (0) | 82 (37.6) |
| LEV | 0.12-16 | 148 (67.8) | 0 (0) | 70 (32.1) |
| CMX | 1-128 | 42 (19.2) | 0 (0) | 177 (81.1) |

PG=Penicillin G, AMC=Amoxicillin-Clavulanic acid, CFM=Cefixime, CTR=Ceftriaxone, VAN=Vancomycin, AZM=Azithromycin, EM=Erythromycin, TET=Tetracycline, GEN=Gentamicin, CM=Clindamycin, NIT=Nitrofurantoin, CIP=Ciprofloxacin, LEV=Levofloxacin, CMX=Co-Trimoxazole, S=Sensitive, I=Intermediate, R=Resistant.

**Table S5. Overall antimicrobial susceptibility profiles of 7 *Bacillus* species**

| **Antibiotic** | **AST of *Bacillus* spp. (%)** | | | | | | |
| --- | --- | --- | --- | --- | --- | --- | --- |
|  | ***B. cereus* (*n*=112)** | ***B. subtilis***  **(*n*=49)** | ***B. amyloliquefaciens* (*n*=20)** | ***B. licheniformis* (*n*=13)** | ***B. thuringiensis* (*n*=11)** | ***B. megaterium* (*n*=8)** | ***B. coagulans* (*n*=5)** |
| PG | S=0 | S=1 (2.0) | S=1 (5.0) | S=0 (0) | S=0 (0) | S=3 (37.5) | S=4 (80.0) |
|  | I=0 | I=0 (0) | I=0 (0) | I=0 (0) | I=0 (0) | I=0 (0) | I=0 (0) |
|  | R=112 (100) | R=48 (98.0) | R=19 (95.0) | R=13 (100) | R=11 (100) | R=5 (62.5) | R=1 (20.0) |
| AMC | S=22 (19.6) | S=18 (36.7) | S=15 (75.0) | S=9 (69.2) | S=6 (54.5) | S=7 (87.5) | S=5 (100) |
|  | I=0 (0) | I=0 (0) | I=0 (0) | I=0 (0) | I=0 (0) | I=0 (0) | I=0 (0) |
|  | R=90 (80.3) | R=31 (63.3) | R=5 (25.0) | R=4 (30.7) | R=5 (45.5) | R=1 (12.5) | R=0 (0) |
| CFM | S=0 (0) | S=0 (0) | S=1 (5.0) | S=0 (0) | S=0 (0) | S=2 (25.0) | S=3(60.0) |
|  | I=0 (0) | I=0 (0) | I=0 (0) | I=0 (0) | I=0 (0) | I=0 (0) | I=0 (0) |
|  | R=112 (100) | R=49 (100) | R=19 (95.0) | R=13 (100) | R=11 (100) | R=6 (75.0) | R=2 (40.0) |
| CTR | S=0 (0) | S=3 (6.1) | S=4 (20.0) | S=2 (15.4) | S=1 (9.0) | S=4 (50.0) | S=4 (80.0) |
|  | I=3 (2.6) | I=7 (14.3) | I=13 (65.0) | I=8 (61.5) | I=5 (45.5) | I=4 (50.0) | I=1 (20.0) |
|  | R=109 (97.4) | R=39 (79.6) | R=3 (15.0) | R=3 (23.1) | R=5 (45.5) | R=0 (0) | R=0 (0) |
| VAN | S=0 (0) | S=1 (2.0) | S=3 (15.0) | S=2 (15.4) | S=1 (9.0) | S=2 (25.0) | S=3 (60.0) |
|  | I=8 (7.2) | I=12 (24.5) | I=15 (75.0) | I=5 (38.5) | I=2 (18.3) | I=6 (75.0) | I=2 (40.0) |
|  | R=104 (92.8) | R=36 (73.5) | R=2 (10.0) | R=6 (46.1) | R=8 (72.7) | R=0 (0) | R=0 (0) |
| AZM | S=18 (16.1) | S=25 (51.0) | S=12 (60.0) | S=8 (61.5) | S=2 (18.2) | S=5 (62.5) | S=3 (60.0) |
|  | I=52 (46.4) | I=20 (40.8) | I=5 (25.0) | I=1 (7.7) | I=3 (27.3) | I=3 (37.5) | I=2 (40.0) |
|  | R=42 (37.5) | R=4 (8.2) | R=3 (15.0) | R=4 (30.8) | R=6 (54.5) | R=0 (0) | R=0 (0) |
| EM | S=5 (4.5) | S=12 (24.5) | S=9 (45.0) | S=6 (46.2) | S=4 (36.3) | S=2 (25.0) | S=3 (60.0) |
|  | I=11 (9.8) | I=30 (61.2) | I=5 (25.0) | I=0 (0) | I=2 (18.2) | I=5 (62.5) | I=2 (40.0) |
|  | R=96 (85.7) | R=7 (14.3) | R=6 (30.0) | R=7 (53.8) | R=5 (45.5) | R=1 (12.5) | R=0 (0) |
| TET | S=8 (7.1) | S=24 (49.0) | S=10 (50.0) | S=3 (23.0) | S=4 (36.3) | S=3 (37.5) | S=4 (80.0) |
|  | I=25 (22.3) | I=21 (42.80) | I=3 (15.0) | I=5 (38.5) | I=2 (18.2) | I=2 (25.0) | I=1 (20.0) |
|  | R=79 (70.5) | R=4 (8.2) | R=7 (35.0) | R=5 (38.5) | R=5 (45.5) | R=3 (37.5) | R=0 (0) |
| GEN | S=112 (100) | S=49 (100) | S=20 (100) | S=13 (100) | S=11 (100) | S=8 (100) | S=5 (100) |
|  | I=0 (0) | I=0 (0) | I=0 (0) | I=0 (0) | I=0 (0) | I=0 (0) | I=0 (0) |
|  | R=0 (0) | R=0 (00 | R=0 (0) | R=0 (0) | R=0 (0) | R=0 (0) | R=0 (0) |
| CM | S=53 (47.3) | S=32 (65.3) | S=12 (60.0) | S=2 (15.4) | S=2 (18.2) | S=8 (100) | S=5 (100) |
|  | I=28 (25.0) | I=10 (20.4) | I=0 (0) | I=4 (30.8) | I=3 (27.3) | I=0 (0) | I=0 (0) |
|  | R=31 (27.7) | R=7 (14.3) | R=8 (40.0) | R=7 (53.8) | R=6 (54.5) | R=0 (0) | R=0 (0) |
| NIT | S=5 (4.5) | S=16 (32.7) | S=5 (25.0) | S=5 (38.5) | S=2 (18.2) | S=8 (100) | S=5 (100) |
|  | I=18 (16.1) | I=20 (40.8) | I=9 (45.0) | I=0 (0) | I=4 (36.3) | I=0 (0) | I=0 (0) |
|  | R=89 (79.4) | R=13 (26.5) | R=6 (30.0) | R=8 (61.5) | R=5 (45.5) | R=0 (0) | R=0 (0) |
| CIP | S=69 (61.6) | S=31 (63.3) | S=13 (65.0) | S=5 (38.5) | S=5 (45.5) | S=8 (100) | S=5 (100) |
|  | I=0 (0) | I=0 (0) | I=0 (0) | I=0 (0) | I=0 (0) | I=0 (0) | I=0 (0) |
|  | R=43 (38.4) | R=18 (36.7) | R=7 (35.0) | R=8 (61.5) | R=6 (54.5) | R=0 (0) | R=0 (0) |
| LEV | S=64 (57.1) | S=42 (85.7) | S=14 (70.0) | S=9 (69.2) | S=6 (54.5) | S=8 (100) | S=5 (100) |
|  | I=0 (0) | I=0 (0) | I=0 (0) | I=0 (0) | I=0 (0) | I=0 (0) | I=0 (0) |
|  | R=48 (42.8) | R=7 (14.3) | R=6 (30.0) | R=4 (30.8) | R=5 (45.5) | R=0 (0) | R=0 (0) |
| CMX | S=3 (2.7) | S=4 (8.2) | S=5 (25.0) | S=8 (61.5) | S=8 (72.7) | S=8 (100) | S=5 (100) |
|  | I=0 (0) | I=0 (0) | I=0 (0) | I=0 (0) | I=0 (0) | I=0 (0) | I=0 (0) |
|  | R=109 (97.3) | R=45 (91.8) | R=15 (75.0) | R=5 (38.5) | R=3 (27.3) | R=0 (0) | R=0 (0) |

PG=Penicillin G, AMC=Amoxicillin-Clavulanic acid, CFM=Cefixime, CTR=Ceftriaxone, VAN=Vancomycin, AZM=Azithromycin, EM=Erythromycin, TET=Tetracycline, GEN=Gentamicin, CM=Clindamycin, NIT=Nitrofurantoin, CIP=Ciprofloxacin, LEV=Levofloxacin, CMX=Co-Trimoxazole, S=Sensitive, I=Intermediate, R=Resistant.

**Table S6. Distributions of antibiotic genes among animal feed, food and diarrhea**

| Antibiotic resistant gene | Animal feed (%) | | | | | | Food (%) | | | Diarrhea (%) | Total (%) (*n*=218) |
| --- | --- | --- | --- | --- | --- | --- | --- | --- | --- | --- | --- |
|  | **LF**  **(*n*=42)** | **BF**  **(*n*=37)** | **DF (*n*=26)** | **CF**  **(*n*=28)** | **FF**  **(*n*=19)** | **Total (*n*=152)** | **E**  **(*n*=25)** | **M**  **(*n*=31)** | **Total**  **(*n*=56)** | **HS**  **(*n*=10)** |  |
| *bla1* | 41 (97.6) | 29 (78.3) | 26 (100) | 13 (46.4) | 14 (73.6) | 123 (80.9) | 7 (28.0) | 17 (54.8) | 24 (42.8) | 9 (90.0) | 156 (71.5) |
| *bla_TEM_* | 7 (16.6) | 3 (8.1) | 6 (23.0) | 2 (7.1) | 3 (15.7) | 21 (13.8) | 4 (16.0) | 4 (12.9) | 8 (14.2) | 1 (10.0) | 30 (13.7) |
| *bla_CTX-M-1_* | 4 (9.5) | 4 (10.8) | 3 (11.5) | 2 (7.1) | 2 (10.5) | 15 (9.8) | 2 (8.0) | 2 (6.4) | 4 (7.1) | 3 (30.0) | 22 (10.1) |
| *bla_CTX-M-2_* | 4 (9.5) | 4 (10.8) | 3 (11.5) | 2 (7.1) | 2 (10.5) | 15 (9.8) | 2 (8.0) | 2 (6.4) | 4 (7.1) | 3 (30.0) | 22 (10.1) |
| *bla_SHV_* | 6 (14.8) | 2 (5.4) | 4 (15.3) | 1 (3.5) | 2 (10.5) | 15 (9.8) | 3 (12.0) | 3 (9.6) | 6 (10.7) | 0 (0) | 21 (9.6) |
| *qnrS* | 0 (0) | 1 (2.7) | 0 (0) | 0 (0) | 1 (5.2) | 2 (1.3) | 5 (20.0) | 2 (6.4) | 7 (12.5) | 0 (0) | 9 (4.1) |
| *sul1* | 1(2.3) | 6 (16.2) | 2 (7.6) | 4 (14.8) | 2 (10.5) | 15 (9.8) | 4 (16.0) | 2 (6.4) | 6 (10.7) | 1 (10.0) | 22 (10.1) |
| *tetA* | 11 (26.1) | 7 (18.9) | 7 (26.9) | 13 (46.4) | 7 (36.8) | 45 (29.6) | 10 (40.0) | 14 (45.1) | 24 (42.8) | 3 (30.0) | 72 (33.0) |
| *erm1* | 11 (26.1) | 6 (16.2) | 11 (42.3) | 4 (14.8) | 6 (31.5) | 36 (23.6) | 9 (36.0) | 11 (35.4) | 20 (35.7) | 6 (60.0) | 59 (27.0) |
| *bla1+bla_TEM_* | 7 (16.6) | 3 (8.1) | 6 (23.0) | 2 (7.1) | 3 (15.7) | 21 (13.8) | 4 (16.0) | 4 (12.9) | 8 (14.2) | 1 (10.0) | 30 (13.7) |
| *bla1+bla_CTX-M-1_* | 3 (7.1) | 3 (8.1) | 1 (3.8) | 1 (3.5) | 1 (5.2) | 9 (5.9) | 1 (4.0) | 1 (3.22) | 2 (3.5) | 2 (20.0) | 13 (5.9) |
| *bla1+bla_CTX-M-2_* | 4 (9.5) | 4 (10.8) | 3 (11.5) | 2 (7.1) | 2 (10.5) | 15 (9.8) | 2 (8.0) | 2 (6.4) | 4 (7.1) | 3 (30.0) | 22 (10.1) |
| *bla1+bla_SHV_* | 6 (14.8) | 2 (5.4) | 4 (15.3) | 1 (3.5) | 2 (10.5) | 15 (9.8) | 3 (12.0) | 3 (9.6) | 6 (10.7) | 0 (0) | 21 (9.6) |
| *bla1+sul-1* | 1 (2.3) | 1 (2.7) | 2 (7.6) | 1 (3.5) | 1 (5.2) | 6 (3.9) | 1 (4.0) | 1 (3.22) | 2 (3.5) | 1 (10.0) | 9 (4.1) |
| *bla1+tetA* | 10 (23.8) | 6 (16.2) | 7 (26.9) | 3 (10.7) | 4 (21.0) | 30 (19.7) | 5 (20.0) | 5 (16.1) | 10 (17.8) | 3 (30.0) | 43 (19.7) |
| *bla1+erm1* | 11 (26.1) | 5 (13.5) | 11 (42.3) | 4 (14.8) | 6 (31.5) | 37 (24.3) | 1 (4.0) | 4 (12.9) | 5 (8.9) | 5 (50.0) | 47 (21.5) |
| *bla_TEM_+bla_CTX-M-1_* | 1 (2.3) | 1 (2.7) | 2 (7.6) | 1 (3.5) | 1 (5.2) | 6 (3.9) | 1 (4.0) | 1 (3.22) | 2 (3.5) | 1 (10.0) | 9 (4.1) |
| *bla_TEM_+bla_CTX-M-2_* | 1 (2.3) | 1 (2.7) | 2 (7.6) | 1 (3.5) | 1 (5.2) | 6 (3.9) | 1 (4.0) | 1 (3.22) | 2 (3.5) | 1 (10.0) | 9 (4.1) |
| *bla_TEM_+bla_SHV_* | 6 (14.8) | 2 (5.4) | 4 (15.3) | 1 (3.5) | 2 (10.5) | 15 (9.8) | 3 (12.0) | 3 (9.6) | 6 (10.7) | 0 (0) | 21 (9.6) |
| *bla_TEM_+su-1* | 1 (2.3) | 1 (2.7) | 2 (7.6) | 1 (3.5) | 1 (5.2) | 6 (3.9) | 1 (4.0) | 1 (3.22) | 2 (3.5) | 1 (10.0) | 9 (4.1) |
| *bla_TEM_+tetA* | 7 (16.6) | 3 (8.1) | 6 (23.0) | 2 (7.1) | 3 (15.7) | 21 (13.8) | 4 (16.0) | 4 (12.9) | 8 (14.2) | 1 (10.0) | 30 (13.7) |
| *bla_TEM_+erm1* | 1 (2.3) | 1 (2.7) | 2 (7.6) | 1 (3.5) | 1 (5.2) | 6 (3.9) | 1 (4.0) | 1 (3.22) | 2 (3.5) | 1 (10.0) | 9 (4.1) |
| *bla_CTX-M-1_+bla_CTX-M-2_* | 4 (9.5) | 4 (10.8) | 3 (11.5) | 2 (7.1) | 2 (10.5) | 15 (9.8) | 2 (8.0) | 2 (6.4) | 4 (7.1) | 3 (30.0) | 22 (10.1) |
| *bla_CTX-M-1_+sul1* | 1 (2.3) | 1 (2.7) | 2 (7.6) | 1 (3.5) | 1 (5.2) | 6 (3.9) | 1 (4.0) | 1 (3.22) | 2 (3.5) | 1 (10.0) | 9 (4.1) |
| *bla_CTX-M-1_+tetA* | 7 (16.6) | 3 (8.1) | 6 (23.0) | 2 (7.1) | 3 (15.7) | 21 (13.8) | 4 (16.0) | 4 (12.9) | 8 (14.2) | 1 (10.0) | 30 (13.7) |
| *bla_CTX-M-1_+erm1* | 1 (2.3) | 1 (2.7) | 2 (7.6) | 1 (3.5) | 1 (5.2) | 6 (3.9) | 1 (4.0) | 1 (3.22) | 2 (3.5) | 1 (10.0) | 9 (4.1) |
| *bla_CTX-M-2_+sul1* | 1 (2.3) | 1 (2.7) | 2 (7.6) | 1 (3.5) | 1 (5.2) | 6 (3.9) | 1 (4.0) | 1 (3.22) | 2 (3.5) | 1 (10.0) | 9 (4.1) |
| *bla_CTX-M-2_+tetA* | 4 (9.5) | 4 (10.8) | 3 (11.5) | 2 (7.1) | 2 (10.5) | 15 (9.8) | 2 (8.0) | 2 (6.4) | 4 (7.1) | 3 (30.0) | 22 (10.1) |
| *bla_CTX-M-2_+erm1* | 1 (2.3) | 1 (2.7) | 2 (7.6) | 1 (3.5) | 1 (5.2) | 6 (3.9) | 1 (4.0) | 1 (3.22) | 2 (3.5) | 1 (10.0) | 9 (4.1) |
| *sul1+tetA* | 1 (2.3) | 1 (2.7) | 2 (7.6) | 1 (3.5) | 1 (5.2) | 6 (3.9) | 1 (4.0) | 1 (3.22) | 2 (3.5) | 1 (10.0) | 9 (4.1) |
| *sul1+erm1* | 1 (2.3) | 1 (2.7) | 2 (7.6) | 1 (3.5) | 1 (5.2) | 6 (3.9) | 1 (4.0) | 1 (3.22) | 2 (3.5) | 1 (10.0) | 9 (4.1) |
| *tetA+erm1* | 1 (2.3) | 1 (2.7) | 2 (7.6) | 1 (3.5) | 1 (5.2) | 6 (3.9) | 6 (24.0) | 6 (19.3) | 12 (21.4) | 1 (10.0) | 19 (8.7) |
| *sul1+tetA+erm1* | 1 (2.3) | 1 (2.7) | 2 (7.6) | 1 (3.5) | 1 (5.2) | 6 (3.9) | 1 (4.0) | 1 (3.22) | 2 (3.5) | 1 (10.0) | 9 (4.1) |
| *bla1*+*bla*_CTX-M-1_+*bla*_CTX-M-2_ | 3 (7.1) | 3 (8.1) | 1 (3.8) | 1 (3.5) | 1 (5.2) | 9 (5.9) | 1 (4.0) | 1 (3.22) | 2 (3.5) | 2 (20.0) | 10 (4.5) |
| *bla1*+*bla*_TEM_+*bla*_CTX-M-1_ | 1 (2.3) | 1 (2.7) | 2 (7.6) | 1 (3.5) | 1 (5.2) | 6 (3.9) | 1 (4.0) | 1 (3.22) | 2 (3.5) | 1 (10.0) | 9 (4.1) |
| *bla1*+*bla*_TEM_+*bla*_SHV_ | 6 (14.8) | 2 (5.4) | 4 (15.3) | 1 (3.5) | 2 (10.5) | 15 (9.8) | 3 (12.0) | 3 (9.6) | 6 (10.7) | 0 (0) | 21 (9.6) |
| *bla1*+*bla*_TEM_+*bla*_CTX-M-1_+*bla*_CTX-M-2_ | 1 (2.3) | 1 (2.7) | 2 (7.6) | 1 (3.5) | 1 (5.2) | 6 (3.9) | 1 (4.0) | 1 (3.22) | 2 (3.5) | 1 (10.0) | 9 (4.1) |
| *bla1*+*bla*_TEM_+*bla*_SHV_+*tetA* | 6 (14.8) | 2 (5.4) | 4 (15.3) | 1 (3.5) | 2 (10.5) | 15 (9.8) | 3 (12.0) | 3 (9.6) | 6 (10.7) | 0 (0) | 21 (9.6) |
| *bla1*+*bla*_CTX-M-1_+*bla*_CTX-M-2_+*tetA* | 3 (7.1) | 3 (8.1) | 1 (3.8) | 1 (3.5) | 1 (5.2) | 9 (5.9) | 1 (4.0) | 1 (3.22) | 2 (3.5) | 2 (20.0) | 13 (5.9) |
| *bla1*+*bla*_TEM_+*bla*_CTX-M-1_+*bla*_CTX-M-2_+*erm1* | 1 (2.3) | 1 (2.7) | 2 (7.6) | 1 (3.5) | 1 (5.2) | 6 (3.9) | 1 (4.0) | 1 (3.22) | 2 (3.5) | 1 (10.0) | 9 (4.1) |
| *bla1*+*bla*_TEM_+*bla*_CTX-M-1_+*bla*_CTX-M-_2+*sul1* | 1 (2.3) | 1 (2.7) | 2 (7.6) | 1 (3.5) | 1 (5.2) | 6 (3.9) | 1 (4.0) | 1 (3.22) | 2 (3.5) | 1 (10.0) | 9 (4.1) |
| *bla1*+*bla*_TEM_+*bla*_CTX-M-1_+*bla*_CTX-M-2_+*tetA*+*erm1* | 1 (2.3) | 1 (2.7) | 2 (7.6) | 1 (3.5) | 1 (5.2) | 6 (3.9) | 1 (4.0) | 1 (3.22) | 2 (3.5) | 1 (10.0) | 9 (4.1) |
| *bla1*+*bla*_TEM_+*bla*_CTX-M-1_+*bla*_CTX-M-2_+*sul1*+*erm1* | 1 (2.3) | 1 (2.7) | 2 (7.6) | 1 (3.5) | 1 (5.2) | 6 (3.9) | 1 (4.0) | 1 (3.22) | 2 (3.5) | 1 (10.0) | 9 (4.1) |
| *bla1*+ *bla*_TEM_+bla_CTX-M-1_+bla_CTX-M-2_+*sul1*+tetA+*erm1* | 1 (2.3) | 1 (2.7) | 2 (7.6) | 1 (3.5) | 1 (5.2) | 6 (3.9) | 1 (4.0) | 1 (3.22) | 2 (3.5) | 1 (10.0) | 9 (4.1) |

LF= layer feed; BF= broiler feed; DF= duck feed; CF= cattle feed; FF= fish feed; E= egg; M= milk; HS= human stool

**Table S7. Prevalence of ARGs of *Bacillus* spp. in animal feed, food and diarrhea**

| ARGs | Occurrence (%) | | | |
| --- | --- | --- | --- | --- |
|  | **Animal feed** | **Food** | **Diarrhea** | **Level of significance** |
|  | **% (n/N)** | **% (n/N)** | **% (n/N)** |  |
| *bla1* | 80.9 (123/152) | 42.8 (24/56) | 90 (9/10) | ** |
| *bla_TEM_* | 13.8 (21/152) | 14.2 (8/56) | 10 (1/10) | NS |
| *bla_CTX-M-1_* | 9.8 (15/152) | 7.1 (4/56) | 30 (3/10) | NS |
| *bla_CTX-M-2_* | 9.8 (15/152) | 7.1 (4/56) | 30 (3/10) | NS |
| *bla_SHV_* | 9.8 (15/152) | 10.7 (6/56) | 0 | NS |
| *qnrS* | 1.3 (2/152) | 12.5 (7/56) | 0 | ** |
| *sul1* | 9.8 (15/152) | 10.7 (6/56) | 10 (1/10) | NS |
| *tetA* | 29.6 (45/152) | 42.8 (24/56) | 30 (3/10) | NS |
| *erm1* | 23.6 (36/152) | 35.7 (20/56) | 60 (6/10) | * |

ARGs=Antibiotic resistance genes, n = Number of resistant genes, N = Number of *Bacillus* isolates. * = significant (*p* < 0.05),

** = significant (*p* < 0.01), NS = non-significant.

**Table S8. Prevalence of ARGs of 7 *Bacillus* species**

| ARGs | Fractions of resistant gene containing isolates | | | | | | | Level of significance |
| --- | --- | --- | --- | --- | --- | --- | --- | --- |
|  | ***B. cereus*** | ***B. subtilis*** | ***B. amyloliquefaciens*** | ***B. licheniformis*** | ***B. thuringiensis*** | ***B. megaterium*** | ***B. coagulans*** |  |
|  | **% (n/N)** | **% (n/N)** | **% (n/N)** | **% (n/N)** | **% (n/N)** | **% (n/N)** | **% (n/N)** |  |
| *bla1* | 70.5 (79/112) | 79.5 (39/49) | 60 (12/20) | 61.5 (8/13) | 63.6 (7/11) | 87.5 (7/8) | 100 (5/5) | NS |
| *bla_TEM_* | 16.9 (19/112) | 0 (0) | 10 (2/20) | 38.4 (5/13) | 27.2 (3/11) | 25 (2/8) | 0 (0) | ** |
| *bla_CTX-M-1_* | 7.1 (8/112) | 4 (2/49) | 5 (1/20) | 15.3 (2/13) | 45.4 (5/11) | 37.5 (3/8) | 0 (0) | ** |
| *bla_CTX-M-2_* | 7.1 (8/112) | 4 (2/49) | 5 (1/20) | 15.3 (2/13) | 45.4 (5/11) | 37.5 (3/8) | 0 (0) | ** |
| *bla_SHV_* | 10.7 (12/112) | 0 (0) | 10 (2/20) | 23 (3/13) | 18.1 (2/11) | 25 (2/8) | 0 (0) | NS |
| *qnrS* | 6.2 (7/112) | 2 (1/49) | 0 (0) | 7.6 (1/13) | 0 (0) | 0 (0) | 0 (0) | NS |
| *sul1* | 6.2 (7/112) | 16.3 (8/49) | 15 (3/20) | 23 (3/13) | 36.3 (4/11) | 0 (0) | 0 (0) | * |
| *tetA* | 40.1 (45/112) | 4 (2/49) | 20 (4/20) | 61.5 (8/13) | 63.6 (7/11) | 62.5 (5/8) | 0 (0) | ** |
| *erm1* | 24.1 (27) | 34.6 (17/49) | 55 (11/20) | 38.4 (5/13) | 9 (1/11) | 12.5 (1/8) | 0 (0) | * |

ARGs=Antibiotic resistance genes, n = Number of resistant genes, N = Number of *Bacillus* isolates, * = significant (*p* < 0.05),

** = significant (*p* < 0.01), NS = non-significant.

**Table S9. Distributions of ARGs among 7 *Bacillus* species**

| ↓Bacterial isolates /ARGs→ | | | Prevalence (%) | | | | | | | | |
| --- | --- | --- | --- | --- | --- | --- | --- | --- | --- | --- | --- |
|  |  |  | ***bla1*** | ***bla*_TEM_** | ***bla*_CTX-M-1_** | ***bla*_CTX-M-2_** | ***bla*_SHV_** | ***qnrS*** | ***sul1*** | ***tetA*** | ***erm1*** |
| *B. cereus*  (*n*=112) | AF (*n*=75) | LF (*n*=22) | 21 (95.4) | 0 (0) | 0 (0) | 0 (0) | 0 (0) | 0 (0) | 0 (0) | 1 (4.5) | 0 (0) |
|  |  | BF (*n*=19) | 18 (94.7) | 1 (5.2) | 1 (5.2) | 1 (5.2) | 0 (0) | 0 (0) | 1 (5.2) | 1 (5.2) | 2 (10.5) |
|  |  | DF (*n*=14) | 14 (100) | 4 (28.5) | 0 (0) | 0 (0) | 4 (28.5) | 0 (0) | 0 (0) | 4 (28.5) | 0 (0) |
|  |  | CF (*n*=12) | 2 (16.6) | 2 (16.6) | 2 (16.6) | 2 (16.6) | 0 (0) | 0 (0) | 2 (16.6) | 12 (100) | 2 (16.6) |
|  |  | FF (*n*=8) | 8 (100) | 3 (37.5) | 1 (12.5) | 1 (12.5) | 2 (25.0) | 0 (0) | 1 (12.5) | 3 (37.5) | 6 (75.0) |
|  |  | Total | 63 (87.5) | 10 (13.3) | 4 (5.3) | 4 (5.3) | 6 (8.0) | 0 (0) | 4 | 21 (28.0) | 10 (13.3) |
|  | ADF (*n*=30) | E (*n*=14) | 4 (28.5) | 4 (28.5) | 1 (7.1) | 1 (7.1) | 3 (21.4) | 5 (45.4) | 1 (7.1) | 9 (64.2) | 6 (42.8) |
|  |  | M (*n*=16) | 5 (31.2) | 4 (25.0) | 2 (12.5) | 2 (12.5) | 3 (18.7) | 2 (12.5) | 1 (6.2) | 14 (87.5) | 6 (37.5) |
|  |  | Total | 9 (30.0) | 8 (26.6) | 3 (10.0) | 3 (10.0) | 6 (20.0) | 7 (23.3) | 2 (6.6) | 23 (76.6) | 12 (40.0) |
|  | *D (n=7)* | HS (*n*=7) | 7 (100) | 1 (14.2) | 1 (14.2) | 1 (14.2) | 0 (0) | 0 (0) | 1 (14.2) | 1 (14.2) | 5 (71.4) |
|  | Total | | 79 (70.5) | 19 (16.9) | 8 (7.1) | 8 (7.1) | 12 (10.7) | 7 (6.2) | 7 (6.2) | 45 (40.1) | 27 (24.1) |
| *B. subtilis*  (*n*=49) | AF (*n*=38) | LF (*n*=12) | 12 (100) | 0 (0) | 2 (16.6) | 2 (16.6) | 0 (0) | 0 (0) | 0 (0) | 2 (16.6) | 10 (83.3) |
|  |  | BF (*n*=9) | 4 (44.4) | 0 (0) | 0 (0) | 0 (0) | 0 (0) | 0 (0) | 5 (55.5) | 0 (0) | 1 (11.1) |
|  |  | DF (*n*=5) | 5 (100) | 0 (0) | 0 (0) | 0 (0) | 0 (0) | 0 (0) | 0 (0) | 0 (0) | 5 (100) |
|  |  | CF (*n*=7) | 5 (71.4) | 0 (0) | 0 (0) | 0 (0) | 0 (0) | 0 (0) | 2 (28.5) | 0 (0) | 0 (0) |
|  |  | FF (*n*=5) | 3 (60.0) | 0 (0) | 0 (0) | 0 (0) | 0 (0) | 1 (20.0) | 1 (20.0) | 0 (0) | 0 (0) |
|  |  | Total | 29 (76.3) | 0 (0) | 2 (5.2) | 2 (5.2) | 0 (0) | 1 (2.6) | 8 (21.0) | 2 (5.2) | 16 (4.1) |
|  | ADF (*n*=8) | E (*n*=0) | 0 (0) | 0 (0) | 0 (0) | 0 (0) | 0 (0) | 0 (0) | 0 (0) | 0 (0) | 0 (0) |
|  |  | M (*n*= 8) | 8 (100) | 0 (0) | 0 (0) | 0 (0) | 0 (0) | 0 (0) | 0 (0) | 0 (0) | 0 (0) |
|  |  | Total | 8 (100) | 0 (0) | 0 (0) | 0 (0) | 0 (0) | 0 (0) | 0 (0) | 0 (0) | 0 (0) |
|  | D (*n*=3) | HS (*n*=3) | 2 (66.6) | 0 (0) | 0 (0) | 0 (0) | 0 (0) | 0 (0) | 0 (0) | 0 (0) | 1 (33.3) |
|  | Total | | 39 (79.5) | 0 (0) | 2 (4.0) | 2 (4.0) | 0 (0) | 1 (2.0) | 8 (16.3) | 2 (4.0) | 17 (34.6) |
| *B. amyloliquefaciens* (*n*=20) | AF (*n*=8) | LF (*n*=1) | 1 (100) | 0 (0) | 1 (100) | 1 (100) | 0 (0) | 0 (0) | 0 (0) | 1 (100) | 0 (0) |
|  |  | BF (*n*=2) | 2 (100) | 2 (100) | 0 (0) | 0 (0) | 2 (100) | 0 (0) | 0 (0) | 2 (100) | 0 (0) |
|  |  | DF (*n*=4) | 4 (100) | 0 (0) | 0 (0) | 0 (0) | 0 (0) | 0 (0) | 0 (0) | 0 (0) | 4 (100) |
|  |  | CF (*n*=0) | 0 (0) | 0 (0) | 0 (0) | 0 (0) | 0 (0) | 0 (0) | 0 (0) | 0 (0) | 0 (0) |
|  |  | FF (*n*=1) | 0 (0) | 0 (0) | 0 (0) | 0 (0) | 0 (0) | 0 (0) | 0 (0) | 1 (100) | 0 (0) |
|  |  | Total | 7 (87.5) | 2 (25.0) | 1 (12.5) | 1 (12.5) | 2 (25.0) | 0 (0) | 0 (0) | 4 (50.0) | 4 (50.0) |
|  | ADF (*n*=12) | E (*n*=8) | 2 (25.0) | 0 (0) | 0 (0) | 0 (0) | 0 (0) | 0 (0) | 3 (37.5) | 0 (0) | 3 (37.5) |
|  |  | M (*n*=4) | 3 (75.0) | 0 (0) | 0 (0) | 0 (0) | 0 (0) | 0 (0) | 0 (0) | 0 (0) | 4 (100) |
|  |  | Total | 5 (41.6) | 0 (0) | 0 (0) | 0 (0) | 0 (0) | 0 (0) | 3 (25.0) | 0 (0) | 7 (58.3) |
|  | D (*n*=0) | HS (0) | 0 (0) | 0 (0) | 0 (0) | 0 (0) | 0 (0) | 0 (0) | 0 (0) | 0 (0) | 0 (0) |
|  | Total | | 12 (60.0) | 2 (10.0) | 1 (5.0) | 1 (5.0) | 2 (10.0) | 0 (0) | 3 (15.0) | 4 (20.0) | 11 (55.0) |
| *B. licheniformis* (*n*=13) | AF (*n*=13) | LF (*n*=3) | 3 (100) | 3 (100) | 1 (33.3) | 1 (33.3) | 2 (66.6) | 0 (0) | 1 (33.3) | 3 (100) | 1 (33.3) |
|  |  | BF (*n*=2) | 0 (0) | 0 (0) | 0 (0) | 0 (0) | 0 (0) | 1 (50.0) | 0 (0) | 1 (50.0) | 0 (0) |
|  |  | DF (*n*=1) | 1 (100) | 1 (100) | 1 (100) | 1 (100) | 0 (0) | 0 (0) | 1 (100) | 1 (100) | 1 (100) |
|  |  | CF (*n*=5) | 4 (80.0) | 1 (20.0) | 0 (0) | 0 (0) | 1 (20.0) | 0 (0) | 1 (20.0) | 1 (20.0) | 3 (60.0) |
|  |  | FF (*n*=2) | 0 (0) | 0 (0) | 0 (0) | 0 (0) | 0 (0) | 0 (0) | 0 (0) | 2 (100) | 0 (0) |
|  |  | Total | 8 (61.5) | 5 (38.4) | 2 (15.3) | 2 (15.3) | 3 (23.0) | 1 (7.6) | 3 (23.0) | 8 (61.5) | 5 (38.4) |
|  | ADF (*n*=0) | E (*n*=0) | 0 (0) | 0 (0) | 0 (0) | 0 (0) | 0 (0) | 0 (0) | 0 (0) | 0 (0) | 0 (0) |
|  |  | M (*n*=0) | 0 (0) | 0 (0) | 0 (0) | 0 (0) | 0 (0) | 0 (0) | 0 (0) | 0 (0) | 0 (0) |
|  |  | Total | 0 (0) | 0 (0) | 0 (0) | 0 (0) | 0 (0) | 0 (0) | 0 (0) | 0 (0) | 0 (0) |
|  | D (*n*=0) | HS (*n*=0) | 0 (0) | 0 (0) | 0 (0) | 0 (0) | 0 (0) | 0 (0) | 0 (0) | 0 (0) | 0 (0) |
|  | Total | | 8 (61.5) | 5 (38.4) | 2 (15.3) | 2 (15.3) | 3 (23.0) | 1 (7.6) | 3 (23.0) | 8 (61.5) | 5 (38.4) |
| *B. thiuringienis* (*n*=11) | AF (*n*=6) | LF (*n*=2) | 2 (100) | 2 (100) | 0 (0) | 0 (0) | 2 (100) | 0 (0) | 0 (0) | 2 (100) | 0 (0) |
|  |  | BF (*n*=3) | 3 (100) | 0 (0) | 3 (100) | 3 (100) | 0 (0) | 0 (0) | 0 (0) | 3 (100) | 0 (0) |
|  |  | DF (*n*=1) | 1 (100) | 1 (100) | 1 (100) | 1 (100) | 0 (0) | 0 (0) | 1 (100) | 1 (100) | 1 (100) |
|  |  | CF (*n*=0) | 0 (0) | 0 (0) | 0 (0) | 0 (0) | 0 (0) | 0 (0) | 0 (0) | 0 (0) | 0 (0) |
|  |  | FF (*n*=0) | 0 (0) | 0 (0) | 0 (0) | 0 (0) | 0 (0) | 0 (0) | 0 (0) | 0 (0) | 0 (0) |
|  |  | Total | 6 (100) | 3 (50.0) | 4 (66.6) | 4 (66.6) | 2 (33.3) | 0 (0) | 1 (16.6) | 6 (100) | 1 (16.6) |
|  | ADF (*n*=5) | E (*n*=3) | 1 (33.3) | 0 (0) | 1 (33.3) | 1 (33.3) | 0 (0) | 0 (0) | 1 (33.3) | 1 (33.3) | 0 (0) |
|  |  | M (*n*=2) | 0 (0) | 0 (0) | 0 (0) | 0 (0) | 0 (0) | 0 (0) | 2 (100) | 0 (0) | 0 (0) |
|  |  | Total | 1 (20.0) | 0 (0) | 1 (20.0) | 1 (20.0) | 0 (0) | 0 (0) | 3 (60.0) | 1 (20.0) | 0 (0) |
|  | D (*n*=0) | HS (*n*=0) | 0 (0) | 0 (0) | 0 (0) | 0 (0) | 0 (0) | 0 (0) | 0 (0) | 0 (0) | 0 (0) |
|  | Total | | 7 (63.6) | 3 (27.2) | 5 (45.4) | 5 (45.4) | 2 (18.1) | 0 (0) | 4 (36.3) | 7 (63.6) | 1 (9.0) |
| *B. megaterium*  (*n*=8) | AF (*n*=8) | LF (*n*=2) | 2 (100) | 2 (100) | 0 (0) | 0 (0) | 2 (100) | 0 (0) | 0 (0) | 2 (100) | 0 (0) |
|  |  | BF (*n*=1) | 0 (0) | 0 (0) | 0 (0) | 0 (0) | 0 (0) | 0 (0) | 0 (0) | 0 (0) | 1 (100) |
|  |  | DF (*n*=1) | 1 (100) | 0 (0) | 1 (100) | 1 (100) | 0 (0) | 0 (0) | 0 (0) | 1 (100) | 0 (0) |
|  |  | CF (*n*=3) | 3 (100) | 0 (0) | 1 (33.3) | 1 (33.3) | 0 (0) | 0 (0) | 0 (0) | 1 (33.3) | 0 (0) |
|  |  | FF (*n*=1) | 1 (100) | 0 (0) | 1 (100) | 1 (100) | 0 (0) | 0 (0) | 0 (0) | 1 (100) | 0 (0) |
|  |  | Total | 7 (87.5) | 2 (25.0) | 3 (37.5) | 3 (37.5) | 2 (25.0) | 0 (0) | 0 (0) | 5 (62.5) | 1 (12.5) |
|  | ADF (*n*=0) | E (*n*=0) | 0 (0) | 0 (0) | 0 (0) | 0 (0) | 0 (0) | 0 (0) | 0 (0) | 0 (0) | 0 (0) |
|  |  | M (*n*=0) | 0 (0) | 0 (0) | 0 (0) | 0 (0) | 0 (0) | 0 (0) | 0 (0) | 0 (0) | 0 (0) |
|  |  | Total | 0 (0) | 0 (0) | 0 (0) | 0 (0) | 0 (0) | 0 (0) | 0 (0) | 0 (0) | 0 (0) |
|  | D (*n*=0) | HS (0) | 0 (0) | 0 (0) | 0 (0) | 0 (0) | 0 (0) | 0 (0) | 0 (0) | 0 (0) | 0 (0) |
|  | Total | | 7 (87.5) | 2 (25.0) | 3 (37.5) | 3 (37.5) | 2 (25.0) | 0 (0) | 0 (0) | 5 (62.5) | 1 (12.5) |
| *B. coagulans*  *(n*=5) | AF (*n*=4) | LF (*n*=0) | 0 (0) | 0 (0) | 0 (0) | 0 (0) | 0 (0) | 0 (0) | 0 (0) | 0 (0) | 0 (0) |
|  |  | BF (*n*=1) | 1 (100) | 0 (0) | 0 (0) | 0 (0) | 0 (0) | 0 (0) | 0 (0) | 0 (0) | 0 (0) |
|  |  | DF (0) | 0 (0) | 0 (0) | 0 (0) | 0 (0) | 0 (0) | 0 (0) | 0 (0) | 0 (0) | 0 (0) |
|  |  | CF (*n*=1) | 1 (100) | 0 (0) | 0 (0) | 0 (0) | 0 (0) | 0 (0) | 0 (0) | 0 (0) | 0 (0) |
|  |  | FF (*n*=2) | 2 (100) | 0 (0) | 0 (0) | 0 (0) | 0 (0) | 0 (0) | 0 (0) | 0 (0) | 0 (0) |
|  |  | Total | 4 (100) | 0 (0) | 0 (0) | 0 (0) | 0 (0) | 0 (0) | 0 (0) | 0 (0) | 0 (0) |
|  | ADF (*n*=1) | E (*n*=0) | 0 (0) | 0 (0) | 0 (0) | 0 (0) | 0 (0) | 0 (0) | 0 (0) | 0 (0) | 0 (0) |
|  |  | M (*n*=1) | 1 (100) | 0 (0) | 0 (0) | 0 (0) | 0 (0) | 0 (0) | 0 (0) | 0 (0) | 0 (0) |
|  |  | Total | 1 (100) | 0 (0) | 0 (0) | 0 (0) | 0 (0) | 0 (0) | 0 (0) | 0 (0) | 0 (0) |
|  | D (n=0) | HS (*n*=0) | 0 (0) | 0 (0) | 0 (0) | 0 (0) | 0 (0) | 0 (0) | 0 (0) | 0 (0) | 0 (0) |
|  | Total | | 5 (100) | 0 (0) | 0 (0) | 0 (0) | 0 (0) | 0 (0) | 0 (0) | 0 (0) | 0 (0) |

AF= Animal feed; ADF= Animal-derived food; D= Diarrhea; LF= Layer feed; BF= Broiler feed; DF= Duck feed; CF= Cattle feed; FF= Fish feed; E= Egg; M= Milk; HS= Human stool

**T****able S10. MDR profiles of *B. cereus*, *B. subtilis*, *B. amyloliquefaciens*, *B. licheniformis*, *B. thuringiensis*, *B. megaterium* and *B. coagulans***

| *B. cereus* | | | | | | | | |
| --- | --- | --- | --- | --- | --- | --- | --- | --- |
| Pattern No. | **Antibiotic resistance pattern** | **No. of antibiotics (classes)** | **Isolates No.** | **Source** | **No. of resistance isolates** | **MDR isolates (%)** | **MAR index** | **Level of significance** |
| 1 | PG-CFM-CTR-CMX | 4 (1) | BC-1, BC-2 | AF | 2 | 0 (0) | 0.28 | ** (*p*=0.000) |
| 2 | PG-CFM-CTR-NIT | 4 (2) | BC-13, BC-14 | AF | 2 | 0 (0) | 0.28 |  |
| 3 | PG-CFM-CTR-TET-NIT | 5 (3) | BC-15 | AF | 1 | 1 (0.89) | 0.35 |  |
| 4 | PG-CFM-CTR-TET-CMX | 5 (3) | BC-3 | AF | 1 | 1 (0.89) | 0.35 |  |
| 5 | PG-CFM-CTR-EM-VAN-CMX | 6 (4) | BC-23, BC-24 | AF | 2 | 2 (1.78) | 0.42 |  |
| 6 | PG-AMC-CFM-CTR-EM-TET-CMX | 7 (4) | BC-4 | AF | 1 | 1 (0.89) | 0.5 |  |
| 7 | PG-AMC-CFM-CTX-EM-VAN-CMX | 7 (4) | BC-65 | AF | 1 | 1 (0.89) | 0.5 |  |
| 8 | PG-AMC-CFM-CTR-VAN-NIT-CMX | 7 (4) | BC-83, BC-84 | F | 2 | 2 (1.78) | 0.5 |  |
| 9 | PG-CFM-CIP-VAN-NIT-CMX | 6 (5) | BC-8 | AF | 1 | 1 (0.89) | 0.42 |  |
| 10 | PG-CFM-CIP-LEV-VAN-NIT-CMX | 7 (5) | BC-9 | AF | 1 | 1 (0.89) | 0.5 |  |
| 11 | PG-CFM-CTR-EM-VAN-NIT-CMX | 7 (5) | BC-37, BC-38 | AF | 2 | 2 (1.78) | 0.5 |  |
| 12 | PG-CFM-CTR-EM-TET-VAN-CMX | 7 (5) | BC-21, BC-22 | AF | 2 | 2 (1.78) | 0.5 |  |
| 13 | PG-AMC-CFM-CTR-EM-TET-VAN-CMX | 8 (5) | BC-5, BC-43, BC-44 | AF | 3 | 3 (2.67) | 0.57 |  |
| 14 | PG-AMC-CFM-CTR-LEV-VAN-NIT-CMX | 8 (5) | BC-28, BC-29 | AF | 2 | 2 (1.78) | 0.57 |  |
| 15 | PG-AMC-CFM-CTR-EM-CM-VAN-CMX | 8 (5) | BC-87 | F | 1 | 1 (0.89) | 0.57 |  |
| 16 | PG-AMC-CFM-CTR-EM-VAN-NIT-CMX | 8 (5) | BC-96, BC-97, BC-98, BC-99, BC-100 | F | 5 | 5 (4.46) | 0.57 |  |
| 17 | PG-AMC-CFM-CTR-TET-VAN-NIT-CMX | 8 (5) | BC-52, BC-53 | AF | 2 | 2 (1.78) | 0.57 |  |
| 18 | PG-AMC-CFM-CTR-TET-LEV-VAN-CMX | 8 (5) | BC-62 | AF | 1 | 1 (0.89) | 0.57 |  |
| 19 | PG-AMC-CFM-CTR-EM-VAN-NIT-CMX | 8 (5) | BC-66, BC-67, BC-68, BC-81, BC-82, BC-85, BC-86 | AF, F | 7 | 7 (6.25) | 0.57 |  |
| 20 | PG-AMC-CFM-CTR-AZM-EM-TET-NIT-CMX | 9 (5) | BC-16 | AF | 1 | 1 (0.89) | 0.64 |  |
| 21 | PG-AMC-CFM-CTR-TET-CIP-LEV-VAN-CMX | 9 (5) | BC-63 | AF | 1 | 1 (0.89) | 0.64 |  |
| 22 | PG-AMC-CFM-CTR-AZM-EM-CM-VAN-CMX | 9 (5) | BC-88, BC-89, BC-90 | F | 3 | 3 (2.67) | 0.64 |  |
| 23 | PG-CFM-CTR-EM-TET-VAN-NIT-CMX | 8 (6) | BC-36, BC-45 | AF | 2 | 2 (1.78) | 0.57 |  |
| 24 | PG-AMC-CFM-CTR-EM-CM-VAN-NIT-CMX | 9 (6) | BC-69, BC-70 | AF | 2 | 2 (1.78) | 0.64 |  |
| 25 | PG-AMC-CFM-CTR-EM-TET-VAN-NIT-CMX | 9 (6) | BC-39, BC-40, BC-41, BC-42, BC-93, BC-94, BC-95, BC-101, BC-102 | AF, F | 9 | 9 (8.03) | 0.64 |  |
| 26 | PG-AMC-CFM-CTR-EM-TET-CIP-LEV-VAN-CMX | 9 (6) | BC-64 | AF | 1 | 1 (0.89) | 0.71 |  |
| 27 | PG-AMC-CFM-CTR-AZM-EM-TET-VAN-NIT-CMX | 10 (6) | BC-91, BC-92, BC-103, BC-104, BC-105, BC-106, BC-107 | D, F | 7 | 7 (6.25) | 0.71 |  |
| 28 | PG-AMC-CFM-CTR-AZM-EM-TET-CIP-LEV-VAN-CMX | 11 (6) | BC-17, BC-18, BC-19, BC-20 | AF | 4 | 4 (3.57) | 0.78 |  |
| 29 | PG-CFM-EM-TET-CIP-VAN-NIT-CMX | 8 (7) | BC-7 | AF | 1 | 1 (0.89) | 0.57 |  |
| 30 | PG-CFM-CTR-EM-TET-LEV-VAN-NIT-CMX | 9 (7) | BC-61 | AF | 1 | 1 (0.89) | 0.64 |  |
| 31 | PG-CFM-CTR-EM-TET-CIP-VAN-NFN-CMX | 9 (7) | BC-6 | AF | 1 | 1 (0.89) | 0.64 |  |
| 32 | PG-AMC-CFM-CTR-EM-TET-CIP-LEV-VAN-NIT-CMX | 11 (7) | BC-10, BC-11, BC-12, BC-30, BC-31 | AF | 5 | 5 (4.46) | 0.78 |  |
| 33 | PG-AMC-CFM-CTR-AZM-EM-TET-LEV-VAN-NIT-CMX | 11 (7) | BC-108, BC-109, BC-110 | D | 3 | 3 (2.67) | 0.78 |  |
| 34 | PG-AMC-CFM-CTR-AZM-EM-TET-CIP-LEV-VAN-NIT-CMX | 12 (7) | BC-25, BC-26, BC-27, BC-32, BC-33, BC-34, BC-35 | AF | 7 | 7 (6.25) | 0.85 |  |
| 35 | PG-CFM-CTR-EM-TET-LEV-CM-VAN-NIT-CMX | 10 (8) | BC-59, BC-60 | AF | 2 | 2 (1.78) | 0.71 |  |
| 36 | PG-CFM-CTR-EM-TET-CIP-LEV-CM-VAN-NIT-CMX | 11 (8) | BC-46 | AF | 1 | 1 (0.89) | 0.78 |  |
| 37 | PG-AMC-CFM-CTR-EM-TET-CIP-LEV-CM-VAN-NIT-CMX | 12 (8) | BC-71, BC-72, BC-73, BC-74, BC-75 | AF | 5 | 5 (4.46) | 0.85 |  |
| 38 | PG-AMC-CFM-CTR-AZM-EM-TET-LEV-CM-VAN-NIT-CMX | 12 (8) | BC-111, BC-112 | D | 2 | 2 (1.78) | 0.85 |  |
| 39 | PG-AMC-CFM-CTR-AZM-EM-TET-CIP-CM-VAN-NIT-CMX | 12 (8) | BC-54, BC-55, BC-56 | AF | 3 | 3 (2.67) | 0.85 |  |
| 40 | PG-AMC-CFM-CTR-AZ-EM-TET-CIP-LEV-CM-VAN-NIT-CMX | 13 (8) | BC-47, BC-48, BC-49, BC-50, BC-51, BC-57, BC-58, BC-76, BC-77, BC-78, BC-79, BC-80 | AF, F | 12 | 12 (10.71) | 0.92 |  |
| Total (Overall MDR isolates) | | | | | 112 | 108 (96.4) |  |  |
| *B. subtilis* | | | | | | | |  |
| 1 | PG-CFM | 2 (1) | BS-23, BS-24, BS-25 | AF | 3 | 0 (0) | 0.14 |  |
| 2 | PG-CFM-CTR-NIT-CMX | 5 (3) | BS-8, BS-9, BS-10 | AF | 3 | 3 (6.12) | 0.35 |  |
| 3 | PG-AMC-CFM-CTR-VAN-CMX | 6 (3) | BS-35, BS-36, BS-37, BS-38, BS-39 | F | 5 | 5 (10.2) | 0.42 |  |
| 4 | PG-AMC-CFM-CTR-VAN-CMX | 6 (3) | BS-11, BS-12, BS-13, BS-14, BS-15, BS-16, BS-17, BS-18, BS-19, BS-20, BS-21, BS-22, BS-47, BS-48, BS-49 | D | 15 | 15 (30.61) | 0.42 |  |
| 5 | PG-CFM-CTR-CIP-NIT-CMX | 6 (4) | BS-4, BS-5, BS-6, BS-7 | AF | 4 | 4 (8.16) | 0.42 |  |
| 6 | PG-CFM-EM-CIP-LEV-VAN | 6 (4) | BS-26 | AF | 1 | 1 (2.04) | 0.42 |  |
| 7 | PG-AMC-CFM-CTR-CIP-VAN-CMX | 7 (4) | BS-31 | AF | 1 | 1 (2.04) | 0.5 |  |
| 8 | PG-AMC-CFM-CTR-CIP-LEV-VAN-CMX | 8 (4) | BS=40, BS-41, BS-42 | F | 3 | 3 (6.12) | 0.5 |  |
| 9 | PG-AMC-CFM-CTR-EM-VAN-CMX | 7 (5) | BS-34 | AF | 1 | 1 (2.04) | 0.5 |  |
| 10 | PG-CFM-TET-CIP-LEV-VAN-CMX | 7 (5) | BS-27 | AF | 1 | 1 (2.04) | 0.5 |  |
| 11 | PG-CFM-CTR-EM-CIP-VAN-CMX | 7 (5) | BS-32, BS-33 | AF | 2 | 2 (4.08) | 0.5 |  |
| 12 | CFM-E-CIP-CM-NIT-CMX | 6 (6) | BS-1 | AF | 1 | 1 (2.04) | 0.42 |  |
| 13 | PG-CFM-EM-CIP-CM-NIT-CMX | 7 (6) | BS-2, BS-3 | AF | 2 | 2 (4.08) | 0.5 |  |
| 14 | PG-CFM-TET-CIP-LEV-VAN-NIT-CMX | 8 (6) | BS-28 | AF | 1 | 1 (2.04) | 0.57 |  |
| 15 | PG-AMC-CFM-CTR-AZM-CM-VAN-CMX | 8 (5) | BS-43, BS-44, BS-45, BS-46 | F | 4 | 4 (8.16) | 0.57 |  |
| 16 | PG-AMC-CFM-CTR-TET-CIP-VAN-NIT-CMX | 9 (6) | BS-30 | AF | 1 | 1 (2.04) | 0.64 |  |
| 17 | PG-AMC-CFM-TET-CIP-LEV-VAN-NIT-CMX | 9 (6) | BS-29 | AF | 1 | 1 (2.04) | 0.64 |  |
| Total (Overall MDR isolates) | | | | | 49 | 46 (93.8) |  |  |
| *B. amyloliquefaciens* | | | | | | | |  |
| 1 | PG-CFM | 2 (1) | BA-13, BA-14, BA-15 | AF | 2 | 0 (0) | 0.14 |  |
| 2 | PG-AMC-CFM-LEV | 4 (2) | BA-11 | AF | 1 | 1 (5) | 0.28 |  |
| 3 | PG-AMC-CFM-LEV-NIT | 5 (3) | BA-12 | AF | 1 | 1 (5) | 0.35 |  |
| 4 | PG-AMC-CFM-LEV-CMX | 5 (3) | BA-9, BA-10 | AF | 1 | 1 (5) | 0.35 |  |
| 5 | PG-CTR-AZM-NIT-CMX | 5 (4) | BA-20 | AF | 2 | 2 (10) | 0.35 |  |
| 6 | PG-CFM-CIP-CM-CMX | 5 (4) | BA-7 | F | 1 | 1 (5) | 0.35 |  |
| 7 | PG-AMC-CFM-CIP-LEV-CM-CMX | 8 (4) | BA-8 | F | 2 | 2 (10) | 0.571 |  |
| 8 | CFM-EM-TET-CM-CMX | 5 (5) | BA-1 | F | 1 | 1 (5) | 0.357 |  |
| 9 | PG-CFM-LEV-VAN-NIT-CMX | 6 (5) | BA-16 | AF | 2 | 2 (10) | 0.428 |  |
| 10 | PG-CFM-EM-TET-CM-CMX | 6 (5) | BA-2, BA-3, BA-4 | F | 3 | 3 (15) | 0.5 |  |
| 11 | PG-CFM-EM-CIP-CM-CMX | 6 (5) | BA-5, BA-6 | F | 1 | 1 (5) | 0.428 |  |
| 12 | PG-CFM-TET-CIP-LEV-VAN-NIT-CMX | 8 (6) | BA-17 | AF | 1 | 1 (5) | 0.571 |  |
| 13 | PG-CFM-CTR-AZM-TET-CIP-NIT-CMX | 8 (6) | BA-18, BA-19 | AF | 2 | 2 (10) | 0.571 |  |
| Total (Over all MDR isolates) | | | | | 20 | 18 (90) |  |  |
| *B. licheniformis* | | | | | | | |  |
| 1 | PG-CFM | 2 (1) | BL-4, BL-5 | AF | 2 | 0 (0) | 0.142 |  |
| 2 | PG-CFM-AZM-EM-CIP-NITCMX | 7 (5) | BL-6 | AF | 1 | 1 (7.69) | 0.5 |  |
| 3 | PG-CFM-AZM-EM-TET-CM-VAN | 7 (5) | BL-1 | AF | 1 | 1 (7.69) | 0.5 |  |
| 4 | PG-CFM-CTR-CIP-LEV-VAN-NIT-CMX | 8 (5) | BL-11 | AF | 1 | 1 (7.69) | 0.571 |  |
| 5 | PG-CFM-CTR-TET-CIP-LEV-VAN-NIT | 8 (5) | BL-12, BL-13 | AF | 2 | 2 (15.38) | 0.571 |  |
| 6 | PG-AMC-CFM-CIP-LEV-CM-NIT-CMX | 8 (5) | BL-10 | AF | 1 | 1 (7.69) | 0.571 |  |
| 7 | PG-CFM-AZM-EM-TET-CM-VAN-CMX | 8 (6) | BL-2, BL-3 | AF | 2 | 2 (15.38) | 0.571 |  |
| 8 | PG-AMC-CFM-EM-CIP-CM-NIT-CMX | 8 (8) | BL-7, BL-8, BL-9 | AF | 3 | 3 (23.07) | 0.571 |  |
| Total (Over all MDR isolates) | | | | | 13 | 11 (84.6) |  |  |
| *B. thuringiensis* | | | | | | | |  |
| 1 | PG-CFM | 2 (1) | BT-4, BT-5 | F | 2 | 0 (0) | 0.142 |  |
| 2 | PG-CFM-TET-CIP-LEV-VAN | 6 (4) | BT-1, BT-2, BT-3 | F | 3 | 3 (27.2) | 0.428 |  |
| 3 | PG-CFM-AZM-CIP-LEV-CM-VAN | 7 (5) | BT-6 | AF | 1 | 1 (9.09) | 0.5 |  |
| 4 | PG-AMC-CFM-CTR-AZM-EM-CM-NIT-CMX | 9 (5) | BT-10 | AF | 1 | 1 (9.09) | 0.642 |  |
| 5 | PG-AMC-CFM-CTR-AZM-EM-CM-VAN-NIT-CMX | 10 (6) | BT-9 | AF | 1 | 1 (9.09) | 0.741 |  |
| 6 | PG-AMC-CFM-CTR-AZM-EM-CM-VAN-NIT-CMX | 10 (6) | BT-11 | AF | 1 | 1 (9.09) | 0.714 |  |
| 7 | PG-AMC-CFM-CTR-AZM-EM-TET-CIP-LEV-CM-VAN-NIT | 12 (7) | BT-7, BT-8 | AF | 2 | 2 (18.2) | 0.857 |  |
| Total (Over all MDR isolates) | | | | | 11 | 9 (81.8) |  |  |
| *B. megaterium* | | | | | | | |  |
| 1 | CFM | 1 (1) | BM-3 | AF | 1 | 0 (0) | 0.071 |  |
| 2 | PG-CFM | 2 (1) | BM-7 | AF | 1 | 0 (0) | 0.142 |  |
| 3 | PG-AMC-CFM | 3 (1) | BM-8 | AF | 1 | 0 (0) | 0.214 |  |
| 4 | PG-CFM-TET | 3 (2) | BM-4, BM-5 | AF | 2 | 0 (0) | 0.214 |  |
| 5 | PG-CFM-EM-TET | 4 (3) | BM-6 | AF | 1 | 1 (16.6) | 0.285 |  |
| Total (Over all MDR isolates) | | | | | 6 | 1 (16.6) |  |  |
| *B. coagulans* | | | | | | | |  |
| 1 | PG | 1 (1) | BCs-4 | AF | 1 | 0 (0) | 0.071 |  |
| 2 | PG-CFM | 2 (1) | BCs-5 | F | 1 | 0 (0) | 0.142 |  |
| Total (Over all MDR isolates) | | | | | 2 | 0 (0) |  |  |

MDR=Multidrug resistance, MAR=Multiple antibody resistance, PG=Penicillin G, AMC=Amoxicillin-Clavulanic acid, CFM=Cefixime, CTR=Ceftriaxone, VAN=Vancomycin, AZM=Azithromycin, EM=Erythromycin, TET=Tetracycline, CM=Clindamycin, NIT=Nitrofurantoin, CIP=Ciprofloxacin, LEV=Levofloxacin, CMX=Co-Trimoxazole

**Table S11. Species wise percentage of MAR index >0.2**

| **Bacterial strains** | **No. of isolates** | **MAR index <0.2** | **MAR index >0.2** | **n/N** | **Percentage** | ***P* value** |
| --- | --- | --- | --- | --- | --- | --- |
| *B*. *cereus* | 112 | 0 | 112 | 112/112 | 100% | 0.000** |
| *B*. *subtilis* | 49 | 3 | 46 | 45/49 | 91.8% |  |
| *B*. *amyloliquefaciens* | 20 | 3 | 17 | 17/20 | 85.0% |  |
| *B*. *licheniformis* | 13 | 2 | 11 | 11/13 | 84.6% |  |
| *B*. *thuringiensis* | 11 | 2 | 9 | 9/11 | 81.8% |  |
| *B*. *megaterium* | 8 | 6 | 2 | 2/8 | 25.0% |  |
| *B*. *coagulans* | 5 | 5 | 0 | 0 | 0 |  |
| Total | 218 | 21 | 197 | 197/218 | 90.3% |  |

N=Number of total isolates, n=Number of isolates having MAR index>0.2, ** Significant at <0.001 level

**Table S12. Sample wise percentage of MAR index >0.2**

| **Sample source** | **No. of isolates** | **MAR index <0.2** | **MAR index >0.2** | **n/N** | **Percentage** | ***P* value** |
| --- | --- | --- | --- | --- | --- | --- |
| Animal feed | 152 | 18 | 134 | 134/152 | 88.1% | 0.213^NS^ |
| Food | 56 | 3 | 53 | 53/56 | 94.6% |  |
| Diarrhea | 10 | 0 | 10 | 10/10 | 100.0% |  |
| Total | 218 | 21 | 197 | 197/218 | 90.3% |  |

N=Number of total isolates, n=Number of isolates having MAR index>0.2, NS= Non-significant

**Table S13. MIC break point of Antibiotic used**

| Category of antibiotic | | MIC range (µg/mL) | MIC break point (µg/mL) | | | Reference |
| --- | --- | --- | --- | --- | --- | --- |
|  |  |  | **S (≤)** | **I** | **R (≥)** |  |
| *Group-I cell wall synthesis inhibitors* | | |  |  |  | CLSI 2020 |
| Beta-lactams | PG | 0.25-32 | 0.25 | - | 32 |  |
|  | AMC | 0.01-0.5 | 0.12/0.06 | - | 0.5/0.25 |  |
|  | CFM | 0.5-8 | 1 | 2 | 4 |  |
|  | CTR | 0.5-8 | 2 | 4 | 8 |  |
| Glycopeptides | VAN | 0.5-64 | 4 | 8-16 | 32 |  |
| *Group-II Protein synthesis inhibitors or DNA damage* | | |  |  |  |  |
| Macrolides | AZM | 0.5-8 | 2 | 4 | 8 |  |
|  | EM | 0.25-32 | 0.5 | 1-4 | 8 |  |
| Tetracyclines | TET | 0.25-32 | 4 | 8 | 16 |  |
| Aminoglycoside | GEN | 0.5-32 | 4 | 8 | 16 |  |
| Lincosamide | CM | 0.25-8 | 0.5 | 1-2 | 4 |  |
| Nitrofuran | NIT | 1-128 | 32 | 64 | 128 |  |
| *Group-III Nucleic acid synthesis inhibitors* | | |  |  |  |  |
| Fluoroquinolones | CIP | 0.12-16 | 1 | 2 | 4 |  |
|  | LEV | 0.12-16 | 1 | 2 | 4 |  |
| Group-IV Folic acid synthesis inhibitors | | |  |  |  |  |
| Sulfonamides | CMX | 1-128 | 2/38 | - | 4/76 |  |

MIC=Minimum inhibitory concentration, PG=Penicillin G, AMC=Amoxicillin-Clavulanic acid, CFM=Cefixime, CTR=Ceftriaxone, VAN=Vancomycin, AZM=Azithromycin, EM=Erythromycin, TET=Tetracycline, GEN=Gentamicin, CM=Clindamycin, NIT=Nitrofurantoin, CIP=Ciprofloxacin, LEV=Levofloxacin, CMX=Co-Trimoxazole, S=Sensitive, I=Intermediate, R=Resistant

**Table S14. PCR protocol of antibiotic resistant gene primer used in this study**

| Target gene | PCR condition | Reference |
| --- | --- | --- |
| *bla1* | 94°C for 5 min; 35 cycles of 94°C for 30 sec, 50°C for 40 sec and 72°C for 45 sec; elongation step at 72 °C for 10 min | Chen et al 2004 |
| *bla_TEM_* | 94°Cfor 3 min; 35 cycles of 94°C for 30 sec, 55°C for 30 sec, and 72°C for 45 sec; elongation step at 72 °C for 5 min | Tokar and Bedenic 2018 |
| *bla*_CTX-M-1_ | 94°C for 5 min; 30 cycles of 94°C for 25 sec, 52°C for 40 sec, and 72°C for 50 sec; elongation step at 72 °C for 6 min |  |
| *bla*_CTX-M-2_ |  |  |
| *bla*_SHV_ | 95°C for 5 min; 30 cycles of 96°C for 30 sec, 62°C for 30 sec, and 72°C for 30 sec; elongation step at 72 °C for 10 min | Adesoji and Ogunjobi et al 2016 |
| *qnrS* | 95°C for 10 min; 35 cycles of 95°C for 1 min, 54°C for 1 min, and 72°C for 1 min; elongation step at 72 °C for 10 min | Cattoir et al 2007 |
| *sul1* | 94°C for 5 min; 30 cycles of 94°C for 30 sec, 57°C for 30 sec, and 72°C for 45 sec; elongation step at 72°C for 7 min | Adekanmbi et al 2020 |
| *tetA* | 94°C for 5 min; 35 cycles of 94°C for 30 sec, 58°C for 40 sec, and 72°C for 45 sec; elongation step at 72 °C for 10 min | Rather et al 2012 |
| *blaA* | 94°C for 5 min; 35 cycles of 94°C for 30 sec, 52°C for 40 sec, and 72°C for 45 sec; elongation step at 72 °C for 10 min | Adimpong et al 2012 |

**Reference**

A.O. Adekanmbi, A.T. Adejoba, O.A. Banjo, M. Saki, Detection of sul1 and sul2 genes in sulfonamide-resistant bacteria (SRB) from sewage, aquaculture sources, animal wastes and hospital wastewater in South-West Nigeria. Gene Rep. 20 (2020) 100742.

A.T. Adesoji, A.A. Ogunjobi, Detection of extended spectrum beta-lactamases resistance genes among bacteria isolated from selected drinking water distribution channels in southwestern Nigeria. BioMed Res. Int. (2016) 7149295.

CLSI. Performance Standards for Antimicrobial Susceptibility Testing, 30th ed. CLSI supplement M100. Wayne, PA: Clinical and Laboratory Standards Institute (2020).

D.B. Adimpong, K.I. Sorensen, L. Thorsen, B. Stuer-Lauridsen, Abdelgadir W.S., Nielsen D.S., et al., Antimicrobial susceptibility of *Bacillus* strains isolated from primary starters for African traditional bread production and characterization of the bacitracin operon and bacitracin biosynthesis. Appl. Environ. Microbiol. 78 (2012) 7903–7914.

K.G. Torkar, B. Bedenić, Antimicrobial susceptibility and characterization of metallo-β-lactamases, extended-spectrum β-lactamases, and carbapenemases of *Bacillus cereus* isolates. Microb. Pathog. 118 (2018) 140-145.

M.A. Rather, R.S. Aulakh, J.P.S. Gill, A.Q. Mir, M.N. Hassan, Detection and sequencing of plasmid encoded tetracycline resistance determinants (tetA and tetB) from food–borne Bacillus cereus isolates. Asian Pac. J. Trop. Med. 5 (2012) 709–712.

N. Sergeev, M. Distler, M. Vargas, V. Chizhikov, K.E. Herold, A. Rasooly. Microarray analysis of *Bacillus* *cereus* group virulence factors. J. Microbiol. Methods 65 (2006), 488–502.

V. Cattoir, L. Poirel, V. Rotimi, C.J. Soussy, P. Nordmann, Multiplex PCR for detection of plasmid-mediated quinolone resistance qnr genes in ESBL-producing enterobacterial isolates. J. Antimicrob. Chemother. 60 (2007) 394–397.

Y. Chen, F.C. Tenover, T.M. Koehler, Beta-lactamase gene expression in a penicillin-resistant *Bacillus anthracis* strain. Antimicrob. Agents Chemother. 48 (2004) 4873-4877.

Z.K. Saeed, B.A. Abbas, R.M. Othman, Molecular identification and phylogenetic analysis of lactic acid bacteria isolated from goat raw milk. Iraqi J. Vet. Sci. 34(2020) 259-263.
